# Using Room Temperature Phosphorescence of Gold(I) Complexes for PAHs Sensing

**DOI:** 10.3390/molecules26092444

**Published:** 2021-04-22

**Authors:** Marian Rosental, Richard N. Coldman, Artur J. Moro, Inmaculada Angurell, Rosa M. Gomila, Antonio Frontera, João Carlos Lima, Laura Rodríguez

**Affiliations:** 1 Department of Inorganic and Organic Chemistry, Inorganic Chemistry Section, Universitat de Barcelona, Martí i Franquès 1, 08028 Barcelona, Spain; Rosental@stud.uni-heidelberg.de (M.R.); richard.n.coldham@gmail.com (R.N.C.); inmaculada.angurell@qi.ub.es (I.A.); 2 Institute of Inorganic Chemistry, Heidelberg University, Im Neuenheimer Feld 270, 69120 Heidelberg, Germany; 3 LAQV-REQUIMTE, Departamento de Química, Universidade Nova de Lisboa, 2829-516 Caparica, Portugal; artur.moro@gmail.com (A.J.M.); lima@fct.unl.pt (J.C.L.); 4 Institut de Nanociència i Nanotecnologia (IN2UB), Universitat de Barcelona, 08028 Barcelona, Spain; 5 Serveis Científico Tècnics, Universitat de les Illes Balears, Crta de Valldemossa km 7.5, 07122 Baleares, Spain; rosa.gomila@uib.es; 6 Departament de Química, Universitat de les Illes Balears, Crta de Valldemossa km 7.5, 07122 Baleares, Spain; toni.frontera@uib.es

**Keywords:** gold(I), polycyclic aromatic hydrocarbons (PAHs), molecular recognition, room temperature phosphorescence, DFT

## Abstract

The synthesis of two new phosphane-gold(I)–napthalimide complexes has been performed and characterized. The compounds present luminescent properties with denoted room temperature phosphorescence (RTP) induced by the proximity of the gold(I) heavy atom that favors intersystem crossing and triplet state population. The emissive properties of the compounds together with the planarity of their chromophore were used to investigate their potential as hosts in the molecular recognition of different polycyclic aromatic hydrocarbons (PAHs). Naphthalene, anthracene, phenanthrene, and pyrene were chosen to evaluate how the size and electronic properties can affect the host:guest interactions. Stronger affinity has been detected through emission titrations for the PAHs with extended aromaticity (anthracene and pyrene) and the results have been supported by DFT calculation studies.

## 1. Introduction

1,8-Naphthalimides (NI) represent a large family of compounds that are easily tunable and highly thermally and chemically stable. Their well-known photophysical properties makes them ideal candidates to be explored in different real-life applications such as the production of organic photochemical dyads and triads, energy storage, light conversion, gels, hybrid materials, optoelectronic devices, solar cells, sensors, biological applications, and bioimaging probes among others [1,2,3,4,5,6,7,8,9].

NI can be easily functionalized allowing not only the possibility to modulate their emission properties on a large scale [1] but also the design of ligands able to coordinate metal atoms giving rise to NI–metal complexes, though these have been less explored than purely organic NI-derivatives. They are also well known to establish π−π intermolecular contacts giving rise to the formation of aggregates, affecting their resulting luminescence in connection with the aggregation induced emission (AIE) and aggregation caused quenching (ACQ) concept [1,6,10,11]. Although fluorescence emission is mainly observed in diluted NI-solution samples, phosphorescence may also be achieved in some cases, such as NI-aggregates or when included within a macrocyclic host [12,13,14]. The heavy atom effect is another way to induce room temperature phosphorescence (RTP) and, to the best of our knowledge, there are very few examples in the literature in the coordination of NI to heavy metals to induce RTP emission and all are platinum derivatives [15,16,17]. 

In this way, gold(I) emerges as a new route to obtain RTP NI-complexes, since it is well known to enhance intersystem crossing and triplet state population [18,19,20,21]. Nevertheless, although there are few examples of gold(I)–NI complexes in the literature, they present fluorescence emission but not phosphorescence [4,22,23,24,25,26].

NI-fluorescence has been used in the molecular recognition of anions, cations and organic molecules [27,28,29,30,31,32]. Changes on the RTP emission band for sensing processes has not been explored in great detail and is a new subject for gold(I)–NI complexes. Hence, the present investigation focuses on the design and synthesis of new 1,8-naphthalimide based gold(I) complexes that display RTP and explores their use in molecular recognition processes. In particular, we are interested in sensing polycyclic aromatic hydrocarbons (PAHs) since they are well-known environmental contaminants with toxic, mutagenic, and carcinogenic properties [33,34].. They can be found in different natural sources such as water and soil and are produced from fossil fuels, industrial manufacturing, residential heating, food preparation, etc. In particular, low molecular weight PAHs (2–3 rings) usually show a higher concentration in water than high molecular weight PAHs (4–6 rings) since they present a higher vapor pressure and solubility in water [34]. Their planar aromatic structure makes them ideal candidates to interact with the NI-core, inducing changes on the emission properties of the gold(I) complexes used as hosts in an easy and fast process. Most importantly, the high sensitivity of luminescence will allow the detection of very low quantities of PAHs.

## 2. Results and Discussion.

### 2.1. Synthesis and Characterization

The synthesis of the two new gold(I) complexes containing a naphthalimide chromophore was performed after previous derivatization of the napthalimide group with a terminal acetylene moiety with slight modifications of the procedure reported in the literature [35]. It is based on a three steps reaction where the 4-bromo-1,8-naphthalic anhydride gives the 6-bromo-1*H*-benzo[de].isoquinoline-1,3(2*H*)-dione (**P1**) that will lead to the final **L** compound through a Sonogashira reaction (Scheme S1).

**Scheme 1:**
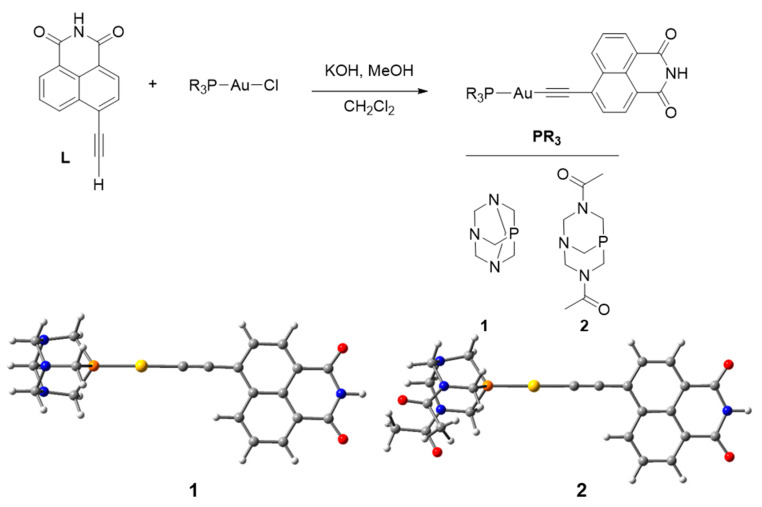
Reaction for the syntheses of the phosphane-gold(I)–napthalimide complexes **1** and **2**.

The deprotonation of the naphthalimide ligand **L** with potassium hydroxide followed by the addition of the stoichiometric amount of [AuCl(PTA)]. or [AuCl(DAPTA)]. (PTA = 1,3,5-Triaza-7-phosphaadamantane; DAPTA = 3,7-Diacetyl-1,3,7-triaza-5-phosphabicyclo[3.3.1].nonane) gold(I) sources yields the desired products **1** and **2** in moderate yield (ca. 50%, Scheme 1).

The absence of the terminal alkynyl C≡C-*H* proton of **L** in the IR and NMR spectra of the complexes confirms the ligand bonds through its alkynyl motif (see Figures S1–S5). A ca. 0.3 ppm upfield shift of naphthalimide signals and the appearance of the expected pattern of the PTA/DAPTA protons in the corresponding ^1^H-NMR spectra confirm the successful synthesis of the complexes. The ^31^P-NMR spectra indicate the presence of a main peak at ca. −49 and −27 ppm for **1** and **2** respectively in accordance with P-donor coordination to the metal center [36,37]. HRESI-MS spectrometry gives the final evidence of the correct formation of the compounds with the corresponding molecular [M + H].^+^ peaks at *m*/*z* 575.0922 and 647.1134 for **1** and **2,** respectively. The presence of broad signals at ^1^H-NMR and additional peaks at ^31^P-NMR spectra suggest possible intermolecular aggregation assemblies, which was confirmed through different methodologies (see below).

### 2.2. Photophysical Characterization

Absorption, emission and excitation spectra of **L** and complexes **1** and **2** were recorded in DMSO at 2 × 10^−5^ M and the results are summarized in Table 1.

**Figure 1:**
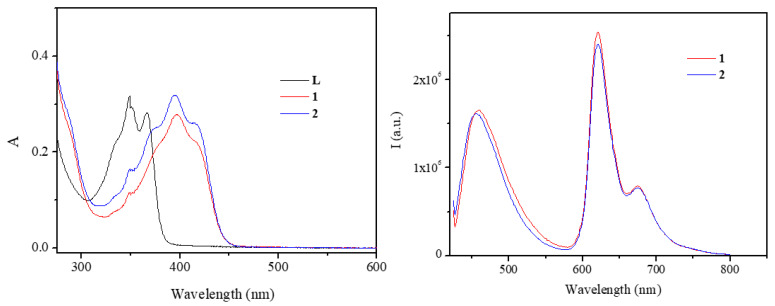
Absorption (**left**) and emission (*λ_exc_* = 418 nm, **right**) spectra of **L** and complexes **1** and **2** in DMSO.

The absorption spectra of the compounds display a vibronically structured band with spacings of 1368 cm^−1^ corresponding to π−π* intraligand transition of the naphthalimide ligand [9,22,24,38,39,40,41]. This band is ca. 50 nm red-shifted upon coordination to the metal atom, as commonly observed for other chromophores coordinated to the Au(I) unit (Figure 1) [36,37]. The emission of **1** and **2** display two bands, one at ca. 455 nm due to the intraligand fluorescence emission of the naphthalimide group and another at longer wavelengths (ca. 620 nm) attributed to the phosphorescence of the chromophore [12,22,23,39,41]. The phosphorescence assignment is evidenced by the quenching of the intensity of this emission band in the presence of oxygen (Figure S10) and can be achieved thanks to the presence of the gold(I) heavy atom that favors the intersystem crossing and population of the emissive triplet state [42]. The heavy atom effect on room temperature phosphorescence of gold(I) naphthalimide complexes is reported herein for the first time although it has previously been observed in platinum complexes, with the similar vibronically structured shape [39]. Previous luminescent studies with other gold(I)–napthalimide complexes contain *N*-substituted napthalimide chromophores and display pure fluorescence emission [22,23,24,25,26]. In our cases, the naphthalimide group does not present any substitution, being thus less bulky. Hence, the resulting higher planarity of the ligand in our systems may favor the approach between different molecules being the chromophore more affected by the presence of the heavy atom of the neighbor molecules, affecting the resulting phosphorescence emission. No significant emission of **L** was recorded in DMSO although it is emissive in CH_2_Cl_2_ (Figure S11).

**Figure 2:**
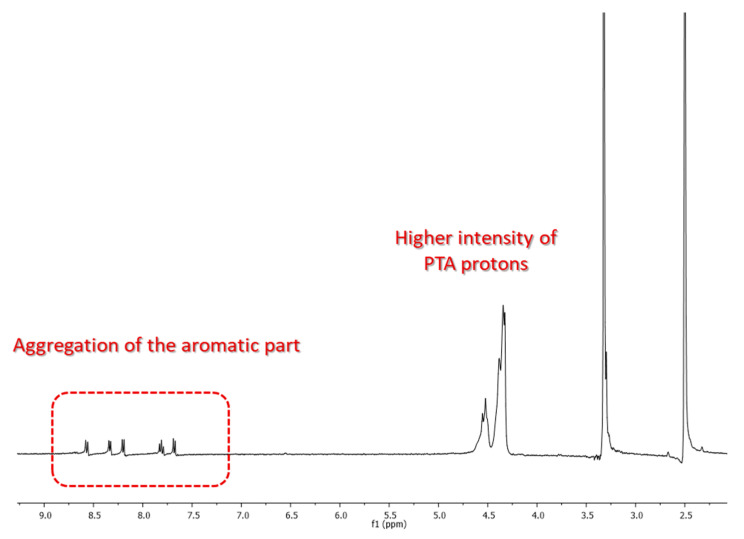
^1^H-NMR spectrum of freshly dissolved solution of **1** in DMSO-*d_6_*.

The room temperature phosphorescence (RTP) of the gold(I) complexes may be observed thanks to the presence of the gold(I) atom in the proximity of the chromophore through intermolecular packing. This is in agreement with the corresponding ^1^H-NMR of freshly prepared samples of **1** and **2** (Figure 2 and Figure S12) which display very low intensity of the aromatic protons, with lower integration values with respect to the rest of the protons of the molecules, the phosphane moieties, as usually happens in this type of gold(I) supramolecular assemblies. Thus this serves as direct evidence of the presence of these aggregation motif [43].

In fact, intermolecular contacts are expected to happen even at lower concentrations than those used for ^1^H-NMR and have been verified to start at concentrations of ca. 2 × 10^−5^ M and 5 × 10^−5^ M (critical aggregation concentrations, c.a.c.) for **1** and **2,** respectively, by recording emission spectra at increasing concentrations (Figures S13 and S14). The lower c.a.c. value of **1** is due to the well-known lower solubility of the PTA phosphane compared to DAPTA. Small well-organized aggregates have been also detected by optical microscopy with cross polarizers (Figure S15).

**Figure 3:**
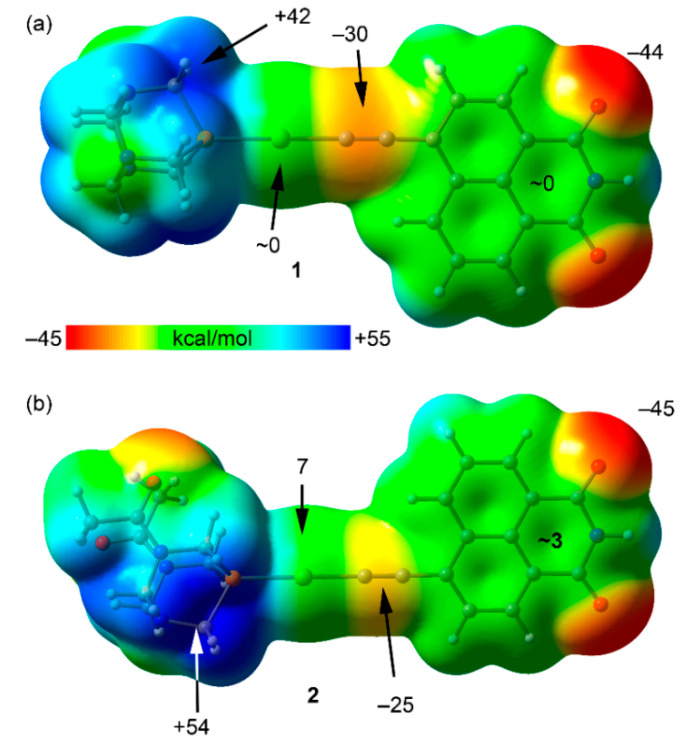
MEP surfaces (isovalue 0.001 a.u.) of compounds **1** (**a**) and **2** (**b**). The values at selected points of the surfaces are indicated in kcal/mol.

The molecular electrostatic potential (MEP) of compounds **1** and **2** have been computed through DFT, to investigate the most electron rich and poor regions of the molecule. The MEP plots are shown in Figure 3, indicating that the most nucleophilic region corresponds to the *O*-atoms of the naphthalimide unit and the most electrophilic one to the *H*-atoms of the phosphine ligands.

The MEP is also negative above and below the triple bond and slightly positive at the Au atom in case of compound **2**. The MEP values are small over the naphthalimide unit.

**Figure 4:**
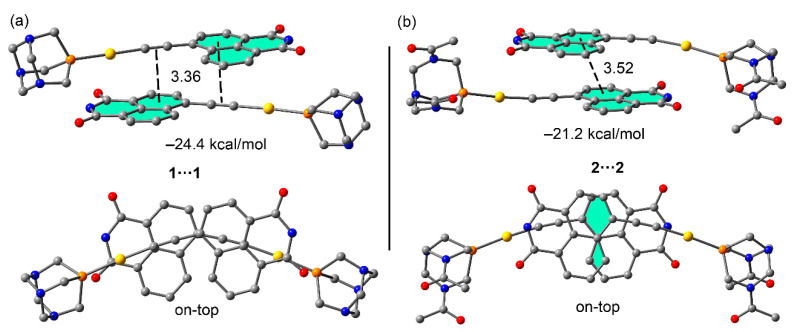
PB86-D3(COSMO)/def2-TZVP optimized geometries of the self-assembled dimers of **1** (**a**) and **2** (**b**). Distances in Å.

The ability for self-assembly and aggregation of compounds **1** and **2** has been computed from the possible self-assembled dimers. The obtained geometries are given in Figure 4 and they adopt an antiparallel arrangement where the naphthalimide unit approaches the phosphane ligand in order to maximize the electrostatic attraction between the negative *O*-atoms of naphthalimide and the positive H-atoms of the Au-coordinated phosphine, in line with the MEP surface analysis. The dimerization energy is larger in compound **1**, in agreement with the larger ability of **1** to form aggregates in DMSO. In the “on-top” representation of the assemblies, it can be observed that there is not π-overlap in compound **1** and, contrariwise, a small overlap between the π-systems of compound **2** is observed.

### 2.3. Molecular Recognition of Polycyclic Aromatic Hydrocarbons, PAHs

**Figure 5:**
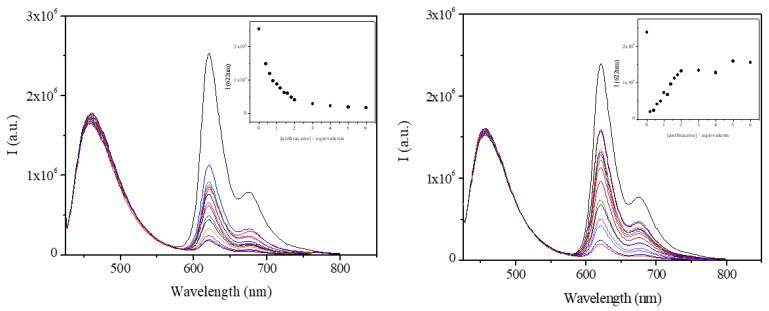
Emission spectra of **1** (**left**) and **2** (**right**) in the presence of different amounts of anthracene. Inset: variations of the emission maxima at 622 nm against [anthracene].

The emissive properties of the gold(I) compounds together with the planarity of the organic chromophore encouraged us to use them as hosts for the molecular recognition of PAHs, being suitable for detection at very low concentrations. With this goal in mind, naphthalene, anthracene, phenanthrene, and pyrene, which differ by the number and position of aromatic rings with an effect on the corresponding size and shape were chosen as target PAHs. Absorption and emission titrations were performed and the changes on the spectroscopic properties of the hosts, **1** and **2**, were followed upon addition of different amounts of the corresponding guest. No significant changes were recorded in any case in the absorption spectra of the host, besides the expected presence of increasing amounts of guest molecules. More interesting data were retrieved from emission titrations. In general trends we can observe the formation of 1:2 host:guest adducts and stronger interactions between the host molecules and PAHs with extended aromaticity present: antracene ≥ pyrene > phenanthrene > naphthalene. Additionally, the resulting adducts with compound **2** are expected to interact more strongly in agreement with the major decrease in the phosphorescence emission intensity recorded (Table 2, Figure 5 and Figures S16–S23). It must be stressed that titrations show, in general, two different tendencies: (i) a (fast) first decrease of the phosphorescence emission (between 0 and 1 equivalent of guest added) and (ii) a recovery of some of the quenched emission reaching a plateau at 2 equivalents of the corresponding guest. Considering that the phosphorescence is expected to be enhanced by the proximity of the chromophore to the gold(I) atom, due to heavy atom effect, the observed variations let us suggest that the interaction with PAHs, especially with the anthracene and pyrene, promotes the disruption of homo-aggregates at low concentration where π···gold or gold···gold interactions are present. At higher concentrations, these interactions are restored to some extent with the formation of hetero-aggregates. The different behaviour in the process between host **1** and anthracene may be ascribed to the phosphorescence recovery at the end of the titration where heteroaggregates are formed from the interaction of the 1:2 adducts as exemplified in Scheme S2. In particular, it may be due to the type of the resulting intermolecular contacts. That is, heteroaggregates containing gold···π or gold···gold interactions in the vicinity of the chromophore are expected to induce the resulting RTP of the naphthalimide. Thus, either anthracene:**1** heterodimers do not form larger heteroaggregates in the concentration range used or the resulting aggregates’ geometry do not contain this type of interactions at this concentration range.

^1^H-NMR experiments did not give structural information of the resulting host:guest adducts since all the host solutions are strongly aggregated at the required NMR concentrations and the peaks are not affected by the addition of the guests (Figures S24–S31).

**Figure 6:**
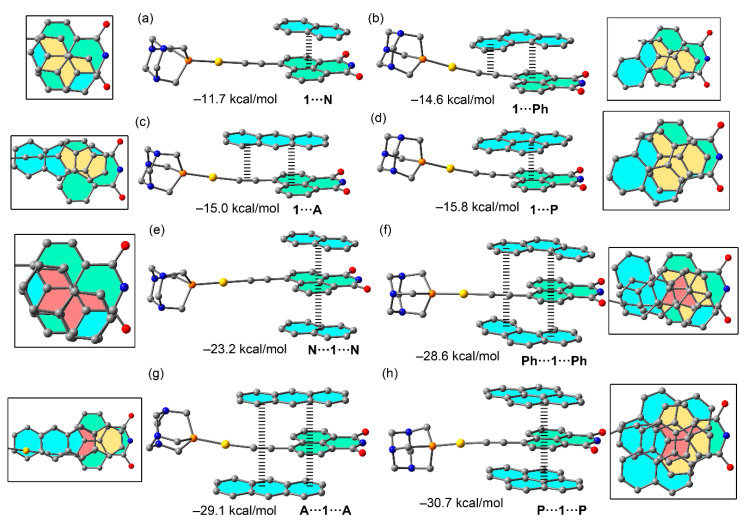
PB86-D3(COSMO)/def2-TZVP optimized geometries and binding energies of the dimers 1···N (**a**), 1···Ph (**b**), 1···A (**c**) 1···P (**d**) and trimers N···1···N (**e**), Ph···1···Ph (**f**), A···1···A (**g**) P···1···P (**h**).

**Figure 7:**
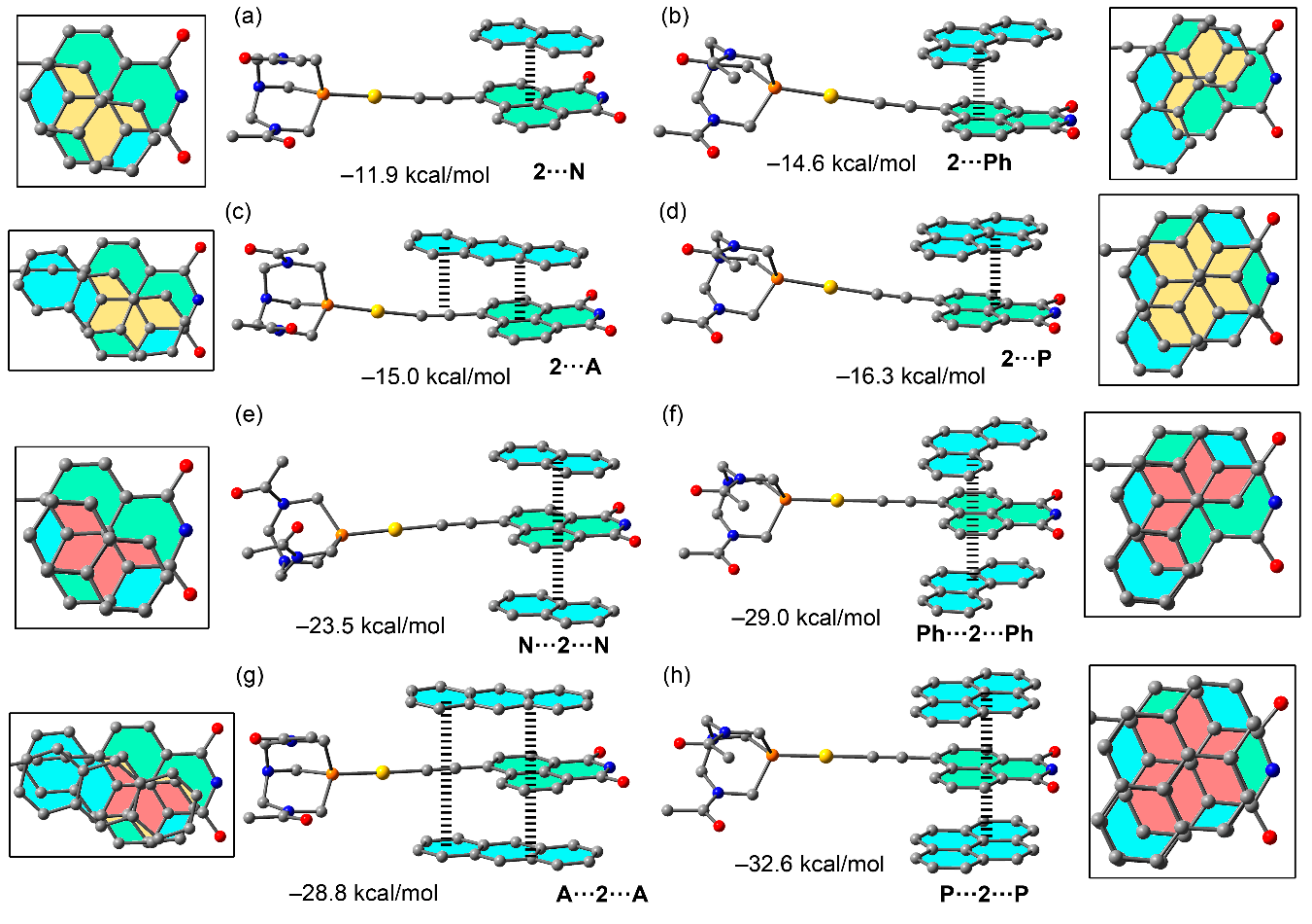
PB86-D3(COSMO)/def2-TZVP optimized geometries and binding energies of the dimers 2···N (**a**), 2···Ph (**b**), 2···A (**c**) 2···P (**d**) and trimers N···2···N (**e**), Ph···2···Ph (**f**), A···2···A (**g**) P···2···P (**h**).

The calculated geometric and energetic features of 1:1 and 1:2 adducts between hosts **1** and **2** and naphthalene (N), anthracene (A), phenanthrene (Ph), and pyrene (P) are shown in Figure 6 and Figure 7, respectively. In all cases the binding site is the naphthalimide that forms stacking interactions with the PAHs. The 1:1 complexes **1**···Ph, **1**···A, and **1**···P exhibit similar interaction energies (−14.6, −15.0, and −15.8 kcal/mol, respectively), which are larger (in absolute value) than that of complex 1···N (−11.7 kcal/mol, respectively). A similar trend is observed in the 1:2 complexes, where Ph, A and P exhibit stronger binding than N. The 1:2 complexes are energetically more favorable than the homodimer **1**···**1** for Ph, A, and P supporting the disaggregation of the host molecules in the presence of these PAHs, as displayed through emission titrations. The opposite is observed for N, in agreement with the absence of interaction detected experimentally for this PAH.

The energies gathered in Figure 6 for the 1:1 and 1:2 complexes suggest that the binding of the first molecule of PAH has little influence on the binding of the second molecule of PAH, thus indicating a lack of cooperativity effects.

The optimized geometries of 1:1 and 1:2 complexes of compound **2** with the PAHs are displayed in Figure 7 and they are similar to those detailed above for compound **1**. The largest 1:1 binding energies are expected for complexes **2**···P, **2**···A, and **2**···Ph with the calculated energies of −16.3, −15.0, and −14.6 kcal/mol, respectively. Again the naphthalene complex is the weakest (−11.9 kcal/mol). A similar trend is observed in the 1:2 complexes.

The reaction energies of the following hypothetical transformations (**H**···**H**) + 2 × PAH → 2 × (**H**···PAH), where **H** stands for hosts **1** and **2** and PAH = N, A, Ph and P, have also been computed. The corresponding reaction energies for such transformations, namely ΔE_d1_ (for **1**) and ΔE_d2_ (for **2**), are gathered in Table 2. That is, assuming that the dimer is the predominant specie in solution, the ΔE_d_ values give hints about the feasibility to transform this strong homodimer (**1**···**1** or **2**···**2**) into two heterodimers upon the addition of one equivalent of PAH (Table 3). It can be observed that in the case of **1** the ΔE_1d_ values suggest that the three larger A, Ph and P are able to form the 1:1 hetero-dimers (exothermic process). In the case of **2**, the ΔE_2d_ values are in all cases negative, thus suggesting that all PAHs should be able to form the host–guest complexes. However, the value is very small for N compared to the others, which is in line with the experimental findings that detect a weak binding for N. In addition, we have also calculated the formation of the heterotrimers using similar transformations: (**H**···**H**) + 4 × PAH → 2 × (PAH···**H**···PAH). The corresponding reaction energies for such transformations, where two equivalents of PAH are added, namely, ΔE_t1_ (for **1**) and ΔE_t2_ (for **2**), are also summarized in Table 2. All reactions are exothermic, thus suggesting than the addition of an excess of PAH should provoke the transformation of the homodimer into the heterotrimer (PAH···**H**···PAH). Inspection of Table 3 lets us to observe a direct trend between ΔE and the number of aromatic rings, with more negative ΔE values as the number of rings increases (2 for N, 3 for Ph and A with similar ΔE, and 4 for P) for both the heterodimers and heterotrimers.

## 3. Conclusions

The self-assembly of phosphane-gold(I)–naphthalimide complexes gives rise to strong aggregates in solution even at very low concentrations (~10^−5^M). Nevertheless, the compounds show a higher affinity to interact with long PAHs compounds, such as anthracene and pyrene. This interaction induces a disassembly of the gold(I) complexes to form very sTable 1:2 (host:guest) adducts. The smaller naphthalene PAH induces little effect on the spectroscopic properties of naphthalimide–gold(I) complexes, indicating that the established π–π interactions with naphthalimide are not strong enough to promote disassembly of these gold(I) complexes.

DFT calculations suggest intermolecular contacts in a head to tail disposition of the gold(I) complexes with Au···π or Au···N-H interactions. The proximity of the gold(I) heavy atom to the naphthalimide chromophore must be the responsible for the observed room temperature phosphorescence emission of the hosts.

## 4. Materials and Methods

### 4.1. General Procedures

All manipulations have been performed under prepurified N_2_ using standard Schlenk techniques. Dry solvents were dispensed from a solvent purification system (Innovative Technologies; Newburyport, MA, USA). Commercial reagents 4-bromo-naphthalic anhydride (Merck, Darmstadt, Germany, 95%), Ammonium hydroxide solution (A.C.S reagent, 28–30%), ethynyltrimethylsilane (Merck, 98%), Copper (I) iodide (Merck, 98%), triethylamine (Merck, 99.5%), 1,3,5-triaza-7-phosphaadamantane (Merck, 97%), tetrabutylammonium fluoride (TBAF, 1M in tetrahydrofuran, Panreac), naphthalene (Koch-Light Labs, Haverhill, UK), anthracene (≥99%, Merck), phenanthrene (≥99.5%, Merck), and pyrene (≥99%, Merck) were used as received.

### 4.2. Physical Measurements

Infrared spectra have been recorded on a FT-IR 520 Nicolet Spectrophotometer. ^1^H NMR (δ(TMS) = 0.0 ppm) and ^31^P{^1^H} NMR (δ(85% H_3_PO_4_) = 0.0 ppm) spectra have been obtained on a Varian Mercury 400, Bruker 400, and Bruker 500. ES(+) mass spectra were recorded on a Fisons VG Quatro spectrometer. Absorption spectra have been recorded on a Varian Cary 100 Bio UV-Spectrophotometer and emission spectra on a Horiba-Jobin-Ybon SPEX Nanolog Spectrofluorimeter.

### 4.3. Theoretical Methods

The calculations of the dimers and trimers were performed at the DFT level of theory using PB86 functional [44] the def2-TZVP basis set [45] and the D3 dispersion correction e4we [46] with the help of the Turbomole 7.0 program package [47]. Solvent effects were considered using COSMO (solvent DMSO) during the optimization [48]. The MEP surfaces were computed at the PB86-D3/def2-TZVP level of theory and visualized using the Gaussview [49] and using the cubes generated by the Multiwfn software [50] and the wavefunction obtained using Turbomole 7.0 (TURBOMOLE GmbH, Karlsruhe, Germany).

### 4.4. Absorption and Emission Titrations

Absorption and emission spectra were recorded using a quartz cuvette with a path length of 10 × 10 mm and a volume of 3 mL. All solutions were prepared with dry DMSO, diluted from a more concentrated stock solution, and used on the same day they were prepared.

### 4.5. Synthesis and Characterization

Synthesis of 6-Bromo-1*H*-benzo[de].isoquinoline-1,3(2*H*)-dione (**P1**)

The synthesis of this compound was adapted from the previous reported in the literature [35].

4-Bromo-1,8-naphthalic anhydride (507 mg, 1.83 mmol, 1 eq.) was suspended in EtOH (10 mL) and 1.50 mL of a 20% NH_3_ solution was added. The mixture was refluxed for 3 h, cooled down to r.t., filtrated and washed with EtOH giving a beige solid (429 mg, 85%). ^1^H-NMR (400 MHz, CDCl_3_): δ = 8.65 (m, *J* = 8.4 Hz, 2H), 8.52 (s, 1H), 8.42 (d, *J* = 7.9 Hz, 1H), 8.08 (d, *J* = 7.9 Hz, 1H), 7.88 (t, *J* = 7.9 Hz, 1H). ESI-MS(+): *m/z* = calc. 275.97 [M + H].^+^, found 275.97.

Synthesis of 6-((Trimethylsilyl)ethynyl)-1*H*-benzo[de].isoquinoline-1,3(2*H*)-dione (**P2**)

6-Bromo-1*H*-benzo[de].isoquinoline-1,3(2*H*)-dione (299 mg, 1.08 mmol, 1.00 eq.) was suspended in dry THF (8 mL) under N_2_ atmosphere and TMS-acetylene (150 mg, 1.53 mmol, 1.40 eq.) in THF (2 mL) was added. CuI (20 mg, 6 mol%) and [PdCl_2_(PPh_3_)_2_]. (18 mg, 3 mmol) were dissolved in dry THF (5 mL) and NEt_3_ (6 mL) was added, then the mixture was added to the previous one and left stirring overnight. DCM (20 mL) was added and the precipitate removed by filtration. The organic layer was washed with sat. NH_4_Cl solution (3 × 10 mL), water (2 × 10 mL), and brine (10 mL), dried over MgSO_4_ and concentrated in vacuum. Purification by column chromatography (SiO_2_, DCM/acetone [10:1]., *v/v*) yielded **P2** as yellow solid (180 mg, 57%). ^1^H-NMR (400 MHz, CDCl_3_): δ = 8.69 (d, *J* = 8.4 Hz, 1H), 8.63 (d, *J* = 7.3 Hz, 1H), 8.52 (dd, *J* = 7.6, 1.1 Hz, 1H), 7.91 (d, *J* = 7.6 Hz, 1H), 7.84 (dd, *J* = 8.2, 7.5 Hz, 1H), 0.37 (s, 9H).

Synthesis of 6-Ethynyl-1*H*-benzo[de].isoquinoline-1,3(2*H*)-dione (**L**)

6-((Trimethylsilyl)ethynyl)-1*H*-benzo[de].isoquinoline-1,3(2*H*)-dione was dissolved in THF (10 mL) and water (1 mL) was added. 1 M TBAF solution in THF (1.25 mL, 1.25 mmol, 2.22 eq.) was added at r.t. and the orange mixture was stirred for 3 h. Water (5 mL) was added and a precipitate formed. The mixture was extracted with DCM (4 × 10 mL) and washed with water (2 × 10 mL) and brine (10 mL), then dried over MgSO_4_. After removing the solvent in vacuum, the solid was washed with hexane and Et_2_O, yielding **L** as pale ochre solid (64 mg, 51%). ^1^H-NMR (400 MHz, CDCl_3_): δ = 8.73 (dd, *J* = 8.4, 1.2 Hz, 1H), 8.64 (dd, *J* = 7.6, 1.2 Hz, 1H), 8.53 (d, *J* = 7.6 Hz, 1H), 8.41 (bs, 1H), 7.97 (d, *J* = 7.6 Hz, 1H), 7.86 (dd, *J* = 8.4, 7.6 Hz, 1H), 3.76 (s, 1H). ^1^H NMR (500 MHz, dmso-d_6_): δ = 11.84 (s, 1H, NH), 8.64 (dd, *J* = 8.4, 1.2 Hz, 1H), 8.51 (dd, *J* = 7.6, 1.2 Hz, 1H), 8.40 (d, *J* = 7.6 Hz, 1H), 8.05 (d, *J* = 7.6 Hz, 1H), 7.98 (dd, *J* = 8.4, 7.6 Hz, 1H), 5.09 (s, 1H). ^13^C-NMR (125 MHz, dmso-d_6_): δ = 164.1(*CO*), 163.8 (*CO*), 131.8 (*CH*), 131.7 (*CH*), 131.6, 130.7, 129.4 (*CH*), 128.7, 128.5 (CH), 125.7, 123.2, 123.0, 90.3 (C*CH*), 80.3(*C*CH). Melting point: 296–298 °C.

Synthesis of [Au(L)(PTA)]. (**1**)

**L** (30 mg, 0.14 mmol, 1.00 eq.) and KOH (40 mg, 0.68 mmol, 5.00 eq.) were suspended in dry MeOH (5 mL) under N_2_ atmosphere and stirred for 30 min. [AuCl(PTA)]. (53 mg, 0.14 mmol, 1.00 eq.) was suspended in dry DCM (5 mL) and added to the other solution leading to the formation of a yellow precipitate. The mixture was stirred overnight, then filtrated through a glass funnel, washed with acetonitrile (10 mL) and Et_2_O (10 mL) and dried in vacuum. [Au(**L**)(PTA)]. (**1**) was obtained as yellow solid (55 mg, 70%). ^1^H-NMR (400 MHz, DMSO-*d*_6_): δ = 8.57 (d, *J* = 8.4 Hz, 1H), 8.33 (d, *J* = 7.2 Hz, 1H), 8.20 (d, *J* = 7.6 Hz, 1H), 7.81 (dd, *J* = 8.4 Hz, *J* = 7.2 Hz, 1H), 7.68 (d, *J* = 7.6 Hz, 1H), 4.52–4.32 (m, 12H). ^31^P NMR (162 MHz, DMSO-*d*_6_): δ = −49.5.0 (s), −20 (s). ESI-MS(+): *m/z* = calc. 575.0866 [M+H].^+^, found 575.0922.

Synthesis of [Au(L)(DAPTA)]. (**2**)

**L** (23 mg, 0.10 mmol, 1.00 eq.) and KOH (19 mg, 0.634 mmol, 3.00 eq.) were suspended in dry MeOH (8 mL) under N_2_ atmosphere and stirred for 30 min. [AuCl(DAPTA)]. (48 mg, 0.10 mmol, 1.00 eq.) was suspended in dry DCM (5 mL) and added to the other solution leading to the formation of a yellow precipitate. The mixture was stirred overnight, then filtrated through a glass funnel, washed with acetonitrile (10 mL) and Et_2_O (10 mL) and dried in vacuum. [Au(**L**)(DAPTA)]. (**2**) was obtained as yellow solid (46 mg, 68%). ^1^H-NMR (400 MHz, DMSO-*d*_6_) δ = 8.63 (d, *J* = 8.0 Hz, 1H), 8.33 (d, *J* = 7.2 Hz, 1H), 8.19 (d, *J* = 7.2 Hz, 1H), 7.80 (dd, *J* = 8.0 Hz, *J* = 7.2 Hz, 1H), 7.64 (d, *J* = 7.2 Hz, 1H), 5.50 (d, *J* = 13.6 Hz, 1H), 5.40 (m, 1H), 4.90 (m, 1H), 4.61 (d, *J* = 14 Hz, 1H), 4.30 (d, *J* = 15.6 Hz, 1H), 4.09 (d, *J* = 14 Hz, 1H), 4.03 (s, 2H), 3.77 (d, *J* = 16 Hz, 1H). ^31^P-NMR (162 MHz, DMSO-*d*_6_) δ = –26.9 (s). ESI-MS(+): *m/z* = calc. 647.1078 [M + H].^+^, found 647.1134.

## Figures and Tables

**Table 1:** Absorption and emission (*λ_exc_* = 418 nm) spectral data of **L**, **1** and **2** in DMSO.

Compound	Absorption $\lambda_{max\;}$(nm) ( $10^{-3}\varepsilon \;$(M^−1^ cm^−1^))	Emission$\;\lambda_{max}\;$(nm)
**L**	367 (14.1), 349 (15.9)	-
**1**	420 (10.5), 397 (13.9), 370 (9.8)	456, 621, 676
**2**	421 (12.3), 396 (15.9), 372 (12.3)	457, 621, 675

**Table 2:** Maximum variations (quenching) on the emission intensity of the host **1** or **2** in the titration with PAHs.

Host/Guest	Anthracene	Naphthalene	Phenanthrene	Pyrene
**1**	85%	<10%	88%	60%
**2**	90%	<10%	90%	90%

**Table 3:** Formation energies of the heterodimers (ΔE_d1_ and ΔE_d2_) and heterotrimers (ΔE_t1_ and ΔE_t2_) in kcal/mol for hosts **1** and **2.**

PAH	Compound 1		Compound 2	
	ΔE_d1_	ΔE_t1_	ΔE_d2_	ΔE_t2_
**N**	+0.8	−22.4	−2.7	−25.8
**Ph**	−4.7	−32.8	−7.9	−36.9
**A**	−5.8	−34.0	−8.8	−36.4
**P**	−7.2	−37.0	−11.5	−43.9

## Data Availability

The data presented in this study are available on request from the corresponding author.

## Supplementary Materials

The following are available, Figure S1: ^1^H NMR spectrum of P1 in CDCl_3_, Figure S2: ^1^H NMR spectrum of P2 in CDCl_3_, Figure S3: ^1^H NMR spectra of L in CDCl_3_ and DMSO-*d*_6_,Figure S4: ^13^C-NMR spectrum of **L** in DMSO-*d*_6_, Figure S5: HSQC NMR spectrum of **L** in DMSO-*d*_6_, Figure S6: ^1^H-NMR spectrum of **1** in DMSO-*d*_6_, Figure S7: ^31^P{^1^H} NMR spectrum of **1** in DMSO-*d*_6_, Figure S8: ^1^H-NMR spectrum of **2** in DMSO-*d*_6_, Figure S9: ^31^P{^1^H} NMR spectrum of **2** in DMSO-*d*_6_, Figure S10: Absorption and emission spectra of **1** and **2** in air-equilibrated and N_2_-saturated solutions, Figure S11: Absorption and Emission spectra of **L** in dichloromethane at 2 × 10^−4^ M concentration (*λ_exc_* = 330 nm), Figure S12: ^1^H-NMR spectrum of freshly dissolved solution of **2** in DMSO-*d*_6_, Figure S13: Emission spectra **1** at different concentrations in DMSO (*λ_exc_* = 370 nm), Figure S14: Emission spectra of **2** at different concentrations in DMSO (*λ_exc_* = 370 nm). Inset: Plot of the intensity of the emission at 435 nm against concentration, Figure S15: Optical microscopy images of **1** (A) and **2** (B) in DMSO at c.a.c., Figure S16: Absorption spectra of **1** in the presence of different amounts of anthracene, Figure S17: Absorption and Emission spectra of **1** in the presence of different amounts of naphthalene. Inset: variations of the emission maxima at 622 nm against [naphthalene], Figure S18: Absorption and Emission spectra of **1** in the presence of different amounts of phenanthrene. Inset: variations of the emission maxima at 622 nm against [phenanthrene], Figure S19: Absorption and Emission spectra of **1** in the presence of different amounts of pyrene. Inset: variations of the emission maxima at 622 nm against [pyrene], Figure S20: Absorption spectra of **2** in the presence of different amounts of anthracene, Figure S21: Absorption and Emission spectra of **2** in the presence of different amounts of naphthalene. Inset: variations of the emission maxima at 622 nm against [naphthalene], Figure S22: Absorption and Emission spectra of **2** in the presence of different amounts of phenanthrene. Inset: variations of the emission maxima at 622 nm against [phenanthrene], Figure S23: Emission spectra of **2** in the presence of different amounts of pyrene. Inset: variations of the emission maxima at 622 nm against [pyrene], Figure S24: ^1^H NMR spectra in DMSO-*d_6_* of **1**, anthracene and 1:1 adduct 1:anthracene, Figure S25: ^1^H NMR spectra in DMSO-*d*_6_ of **1**, naphthalene and 1:1 adduct 1: naphthalene, Figure S26: ^1^H NMR spectra in DMSO-*d*_6_ of **1**, pyrene and 1:1 adduct 1: pyrene, Figure S27: ^1^H NMR spectra in DMSO-*d*_6_ of **1**, phenanthrene and 1:1 adduct 1: phenanthrene, Figure S28: ^1^H NMR spectra in DMSO-*d*_6_ of **2**, anthracene and 1:1 adduct 2:anthracene, Figure S29: ^1^H-NMR spectra in DMSO-*d*_6_ of **2**, naphthalene and 1:1 adduct 2: naphthalene, Figure S30: ^1^H NMR spectra in DMSO-*d*_6_ of **2**, pyrene and 1:1 adduct 2: pyrene, Figure S31: ^1^H NMR spectra in DMSO-*d*_6_ of **2**, phenantrene and 1:1 adduct 2: phenantrene, Scheme S1: Experimental procedure for the synthesis of **L**, Scheme S2: Schematic representation of the possible rationalization of the different steps of the host:guest interaction and resulting aggregates.

## Sample Availability

Samples of the compounds **L**, **1** and **2** are available from the authors.
