# Supplementary material for: Using Room Temperature Phosphorescence of Gold(I) Complexes for PAHs Sensing
Source: Molecules. 2021 Apr 22;26(9):2444. doi: 10.3390/molecules26092444 (PMC8122727; doi:10.3390/molecules26092444)
Supplement: Supplementary file 1 [file molecules-26-02444-s001.zip › molecules-1182626-supplementary.pdf]

Using room temperature phosphorescence of gold(I) complexes for PAHs sensing.

Marian Rosental,[a,b] Richard N. Coldman,[a] Artur J. Moro,[c] Inmaculada Angurell,[a,d] Rosa M Gomila,[e] Antonio Frontera,[f] João Carlos Lima[c] and Laura Rodríguez.[a,d,*]

[a] *Departament de Química Inorgànica i Orgànica. Secció de Química Inorgànica. Universitat de Barcelona, Martí i Franquès 1-11, 08028 Barcelona, Spain. E-mail: laura.rodriguez@qi.ub.es*

[b] *Institute of Inorganic Chemistry, Heidelberg University, Im Neuenheimer Feld 270, 69120 Heidelberg (Germany)*

[c] *LAQV-REQUIMTE, Departamento de Química, Universidade Nova de Lisboa, Monte de Caparica*

[d] *Institut de Nanociència i Nanotecnologia (IN^{2}UB). Universitat de Barcelona, 08028 Barcelona (Spain)*

[e] *Serveis Científico Tècnics, Universitat de les Illes Balears, Crta de Valldemossa km 7.5, 07122 Palma de Mallorca, Baleares, Spain*

[f] *Departament de Química, Universitat de les Illes Balears, Crta de Valldemossa km 7.5, 07122 Palma de Mallorca, Baleares, Spain*

Supporting Information

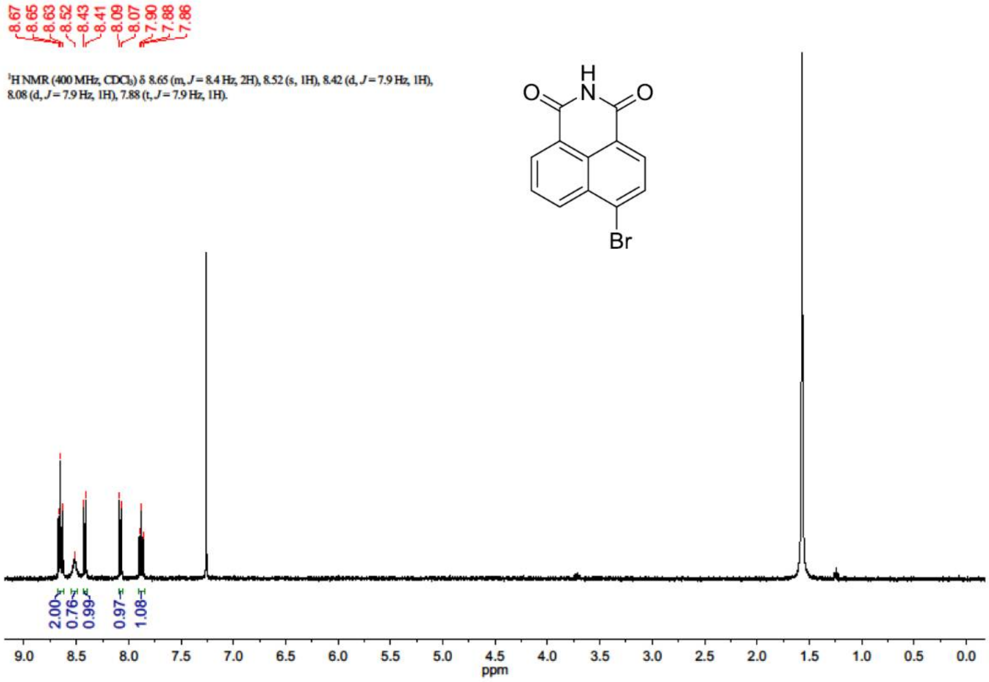

Figure S1. ^{1}H NMR spectrum of **P1** in $CDCl_3$.

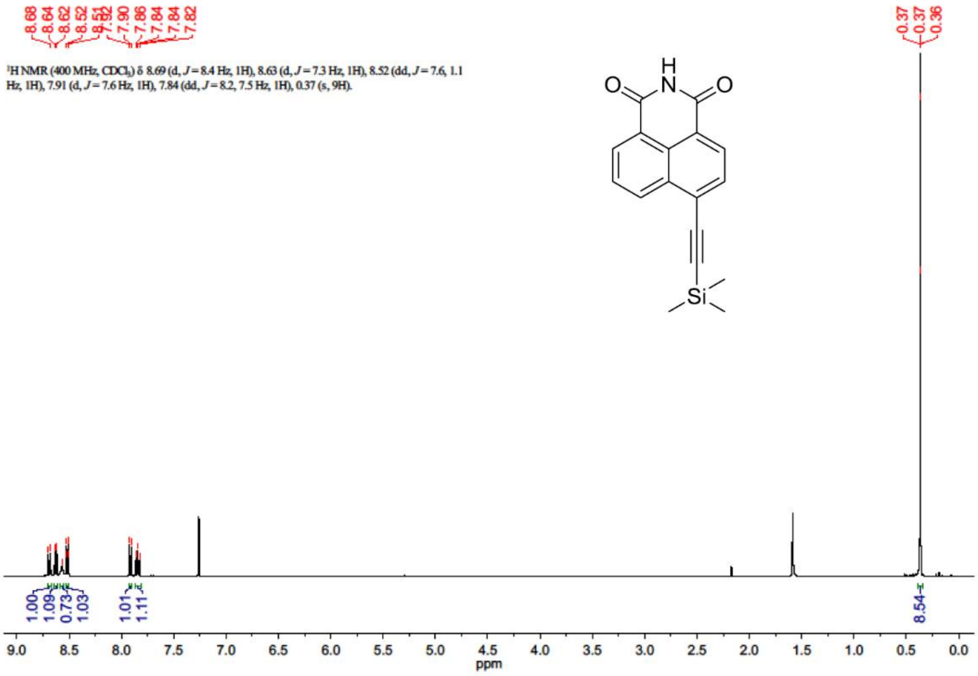

Figure S2. ^{1}H NMR spectrum of **P2** in $CDCl_3$.

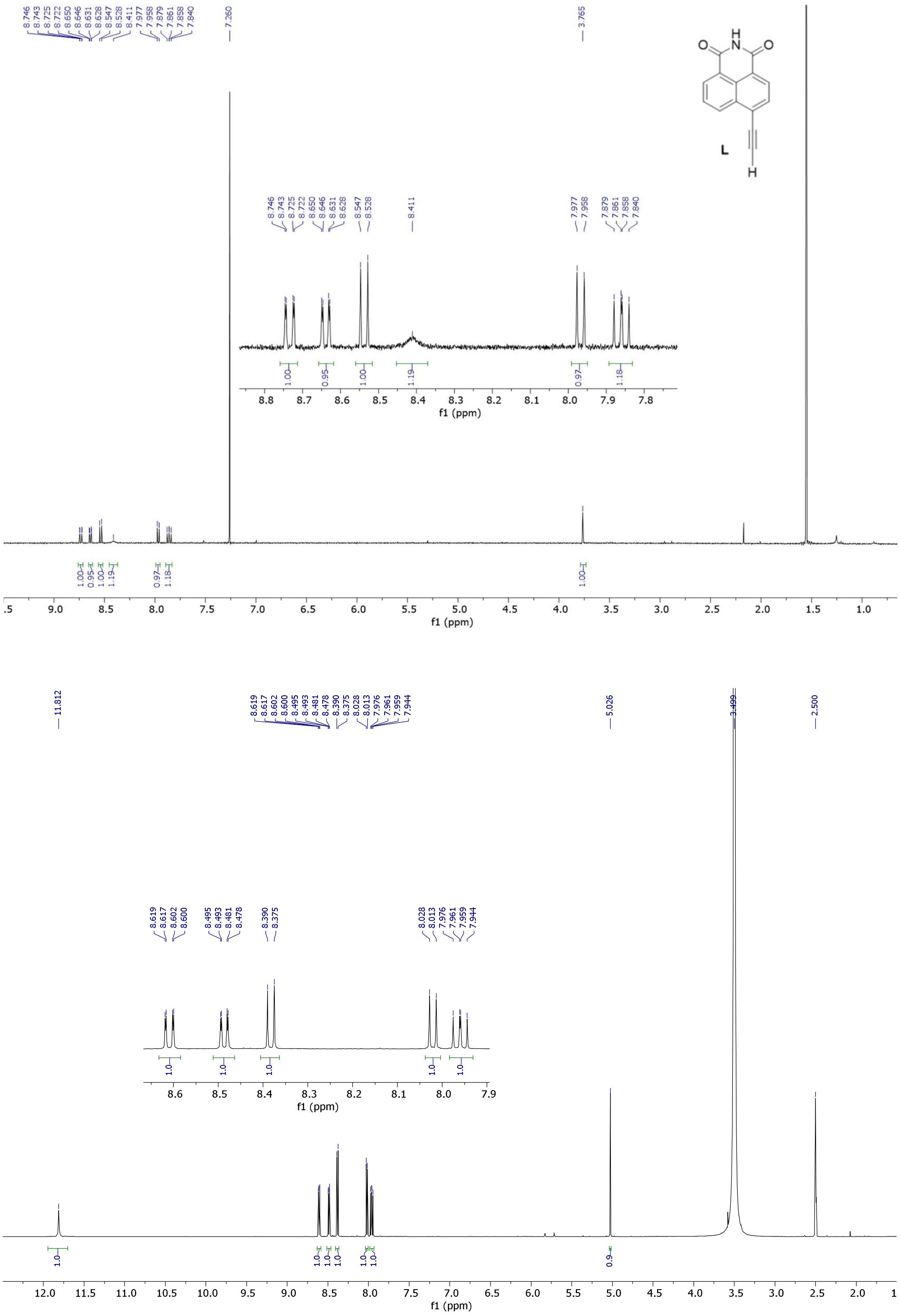

Figure S3. ^{1}H NMR spectrum of **L** in $CDCl_3$ (above) and DMSO-d_6 (bellow).

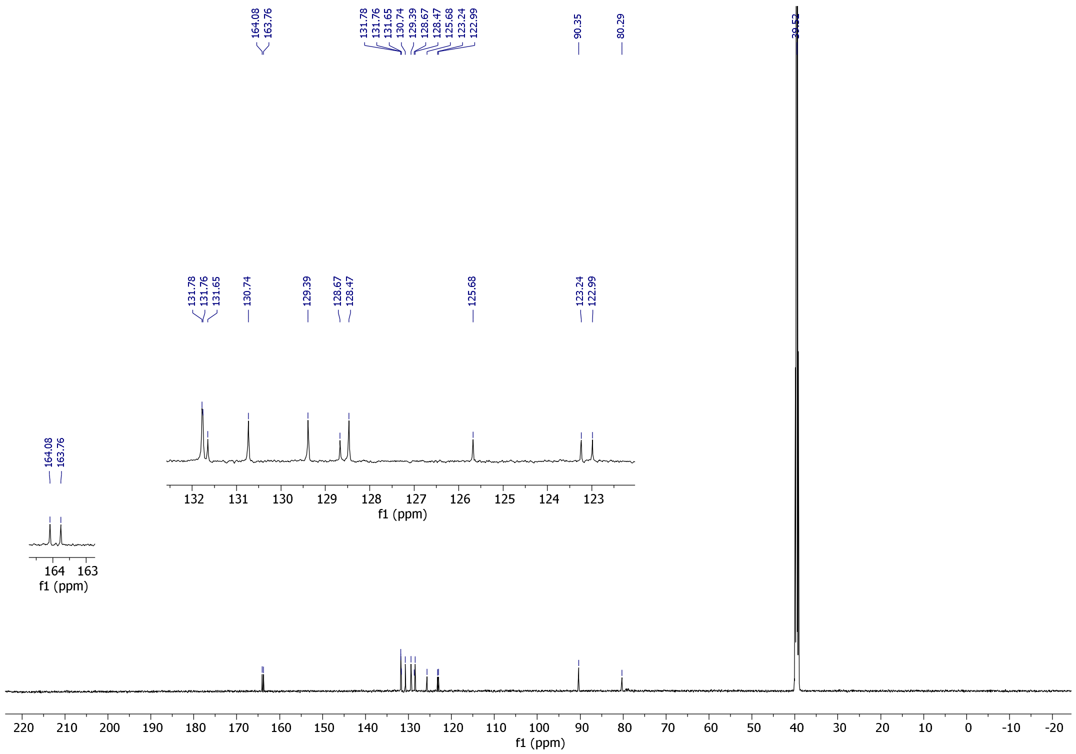

Figure S4. ^{13}C NMR spectrum of **L** in DMSO-d_6.

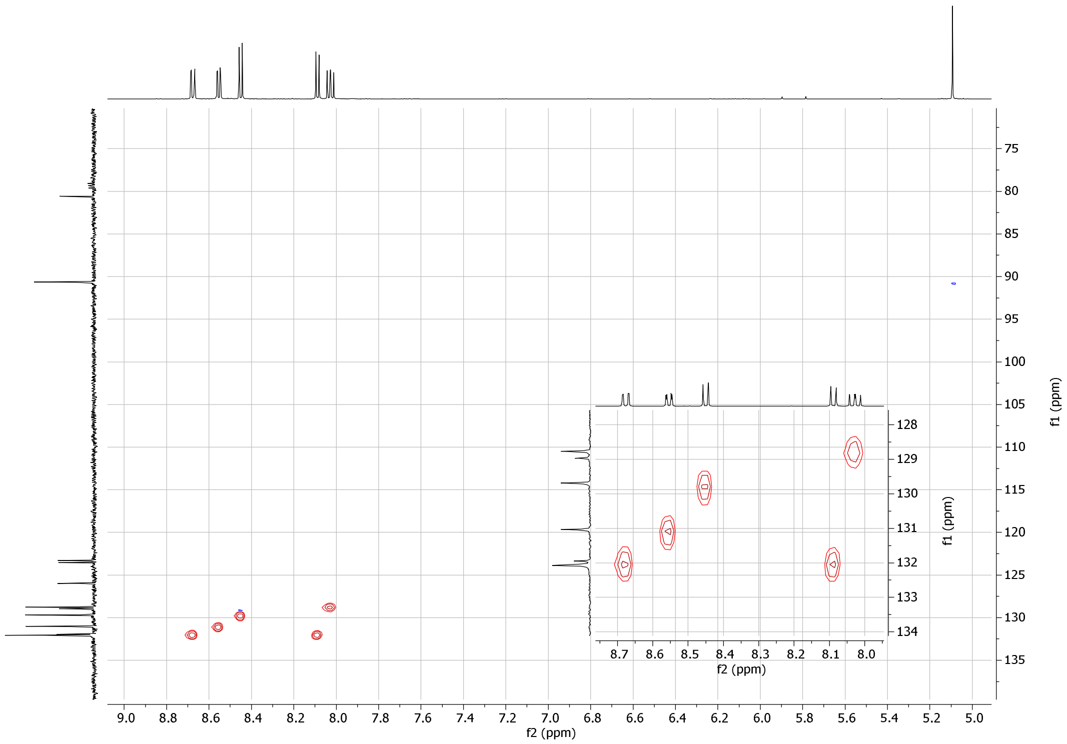

Figure S5. HSQC NMR spectrum of **L** in DMSO-d_6.

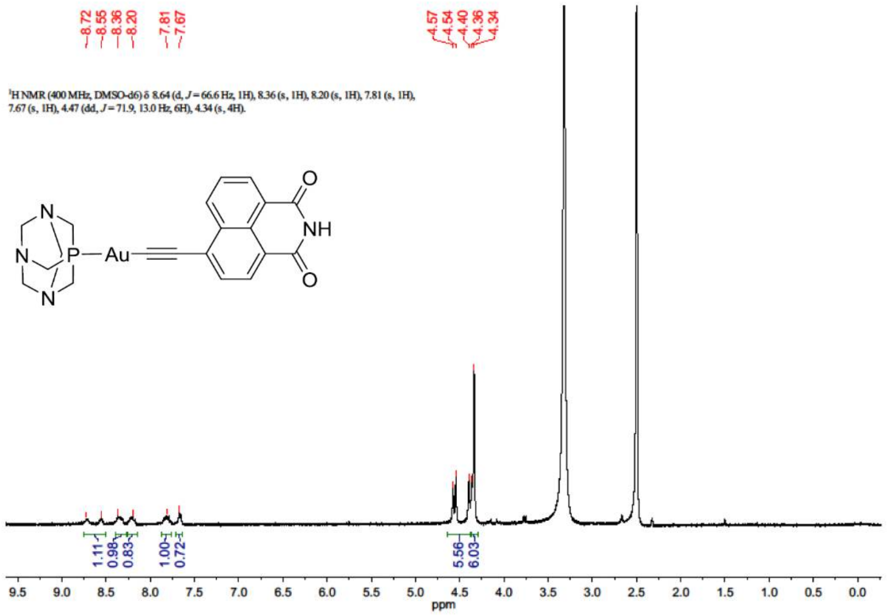

Figure S6. ^{1}H NMR spectrum of **1** in DMSO-d_6.

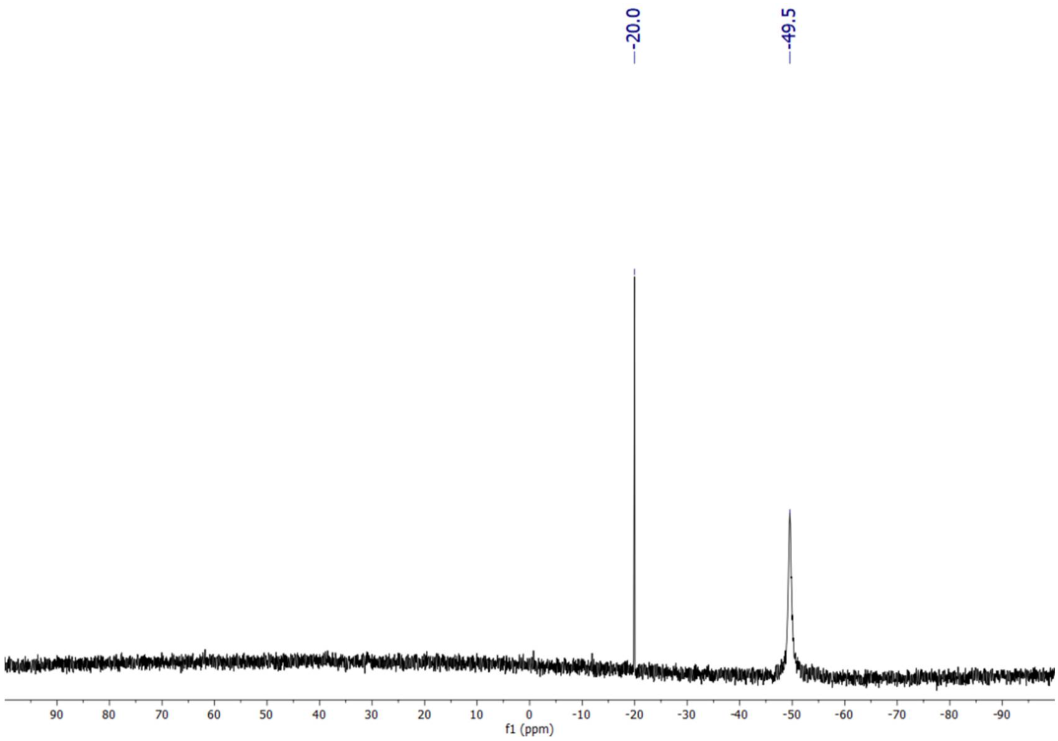

Figure S7. ^{31}P{^{1}H} NMR spectrum of **1** in DMSO-d_6.

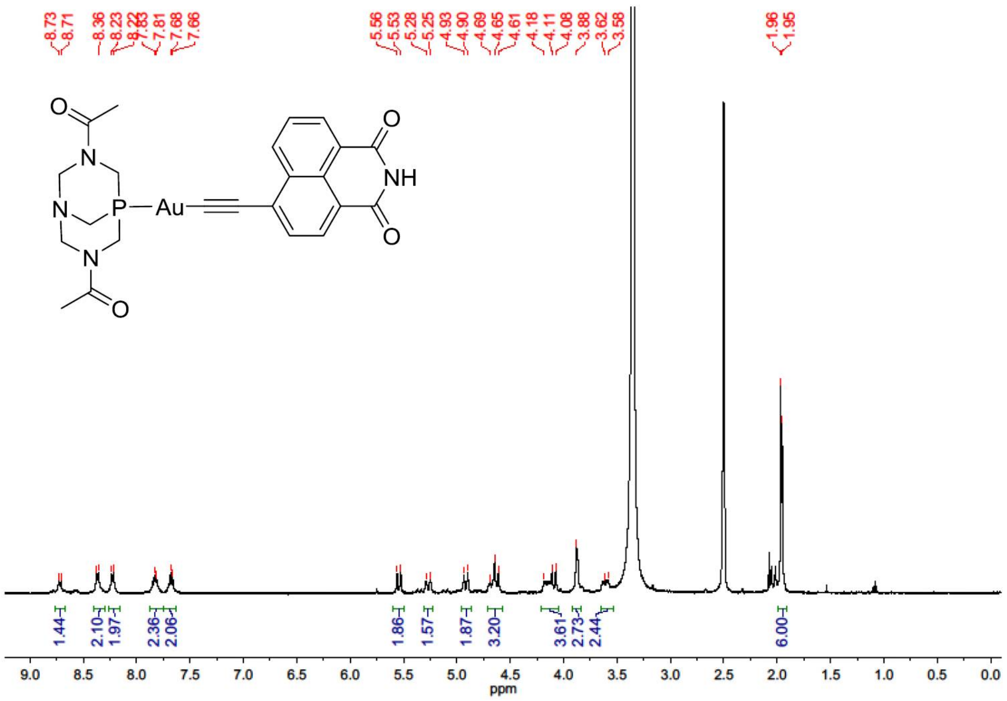

Figure S8. ^{1}H NMR spectrum of **2** in DMSO-d_6.

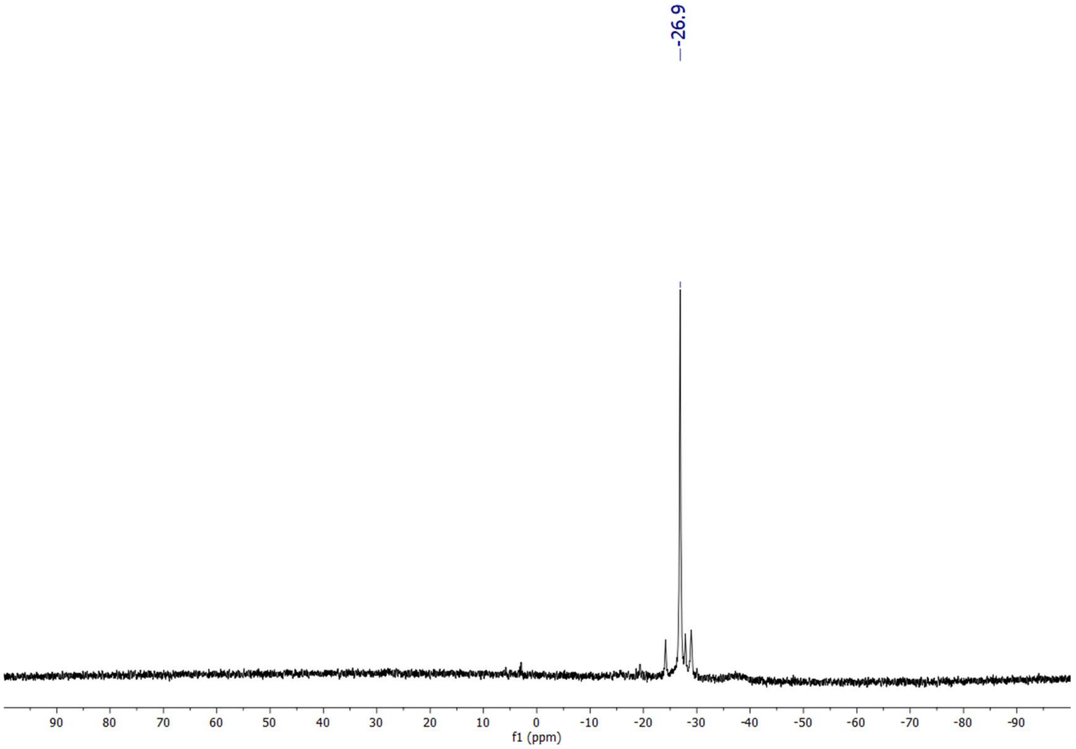

Figure S9. ^{31}P{^{1}H} NMR spectrum of **2** in DMSO-d_6

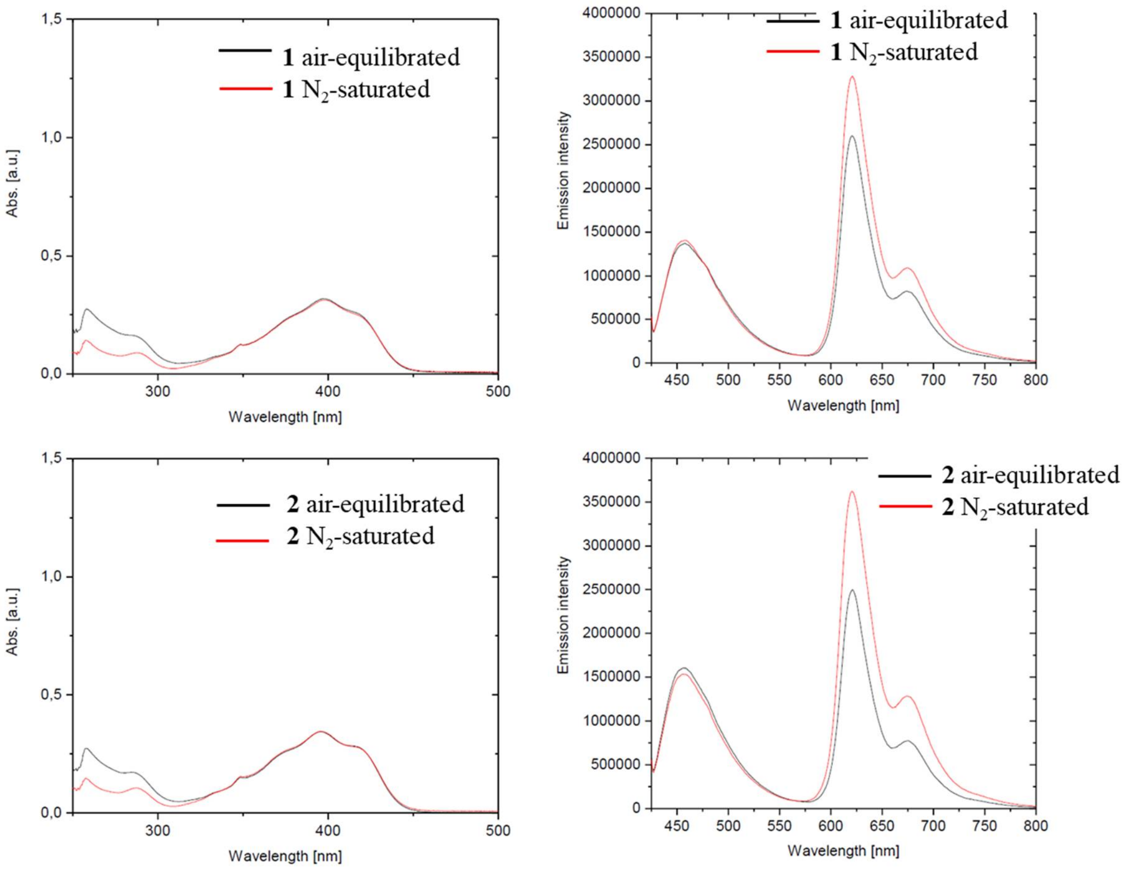

Figure S10. Absorption (left) and emission (right) spectra of **1** (above) and **2** (below) in air-equilibrated and N_2-saturated solutions.

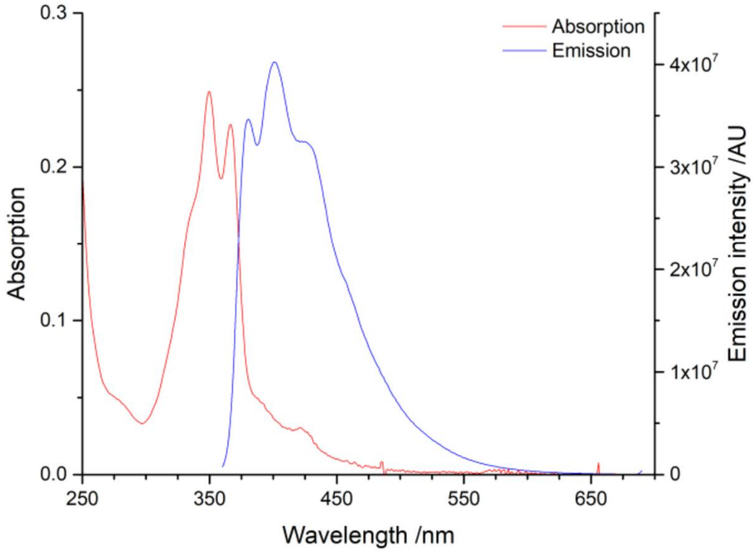

Figure S11. Absorption (red line) and Emission (blue line) spectra of **L** in dichloromethane at $2x10^{-4}$M concentration (λ_{exc} = 330 nm).

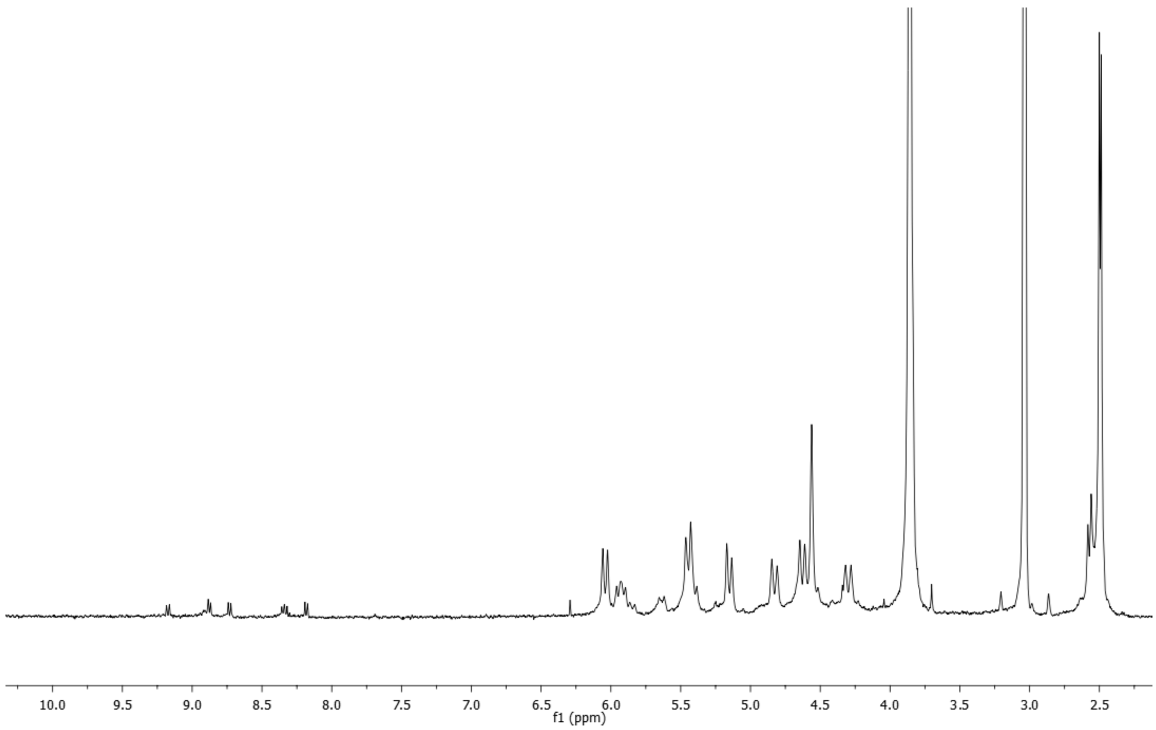

Figure S12. ^{1}H NMR spectrum of freshly dissolved solution of **2** in DMSO-d_6.

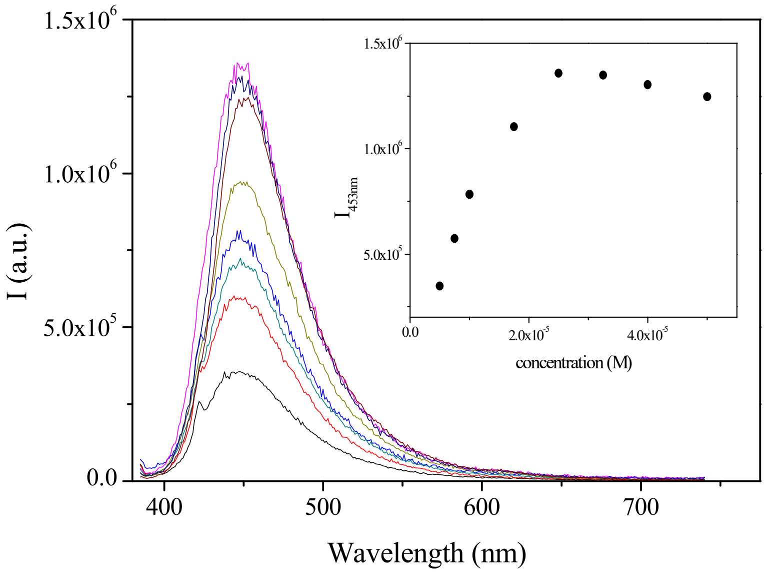

Figure S13. Emission spectra **1** at different concentrations in DMSO (λ_{exc} = 370 nm). Inset: Plot of the intensity of the emission at 453 nm against concentration. Samples sonicated before recording emission spectra (phosphorescence band disappears).

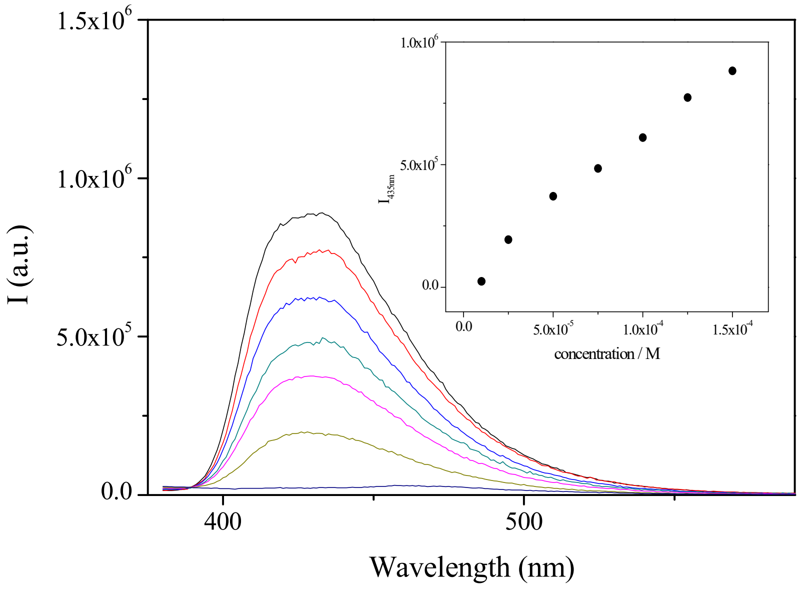

Figure S14. Emission spectra of **2** at different concentrations in DMSO (λ_{exc} = 370 nm). Inset: Plot of the intensity of the emission at 435 nm against concentration. Samples sonicated before recording emission spectra (phosphorescence band disappears).

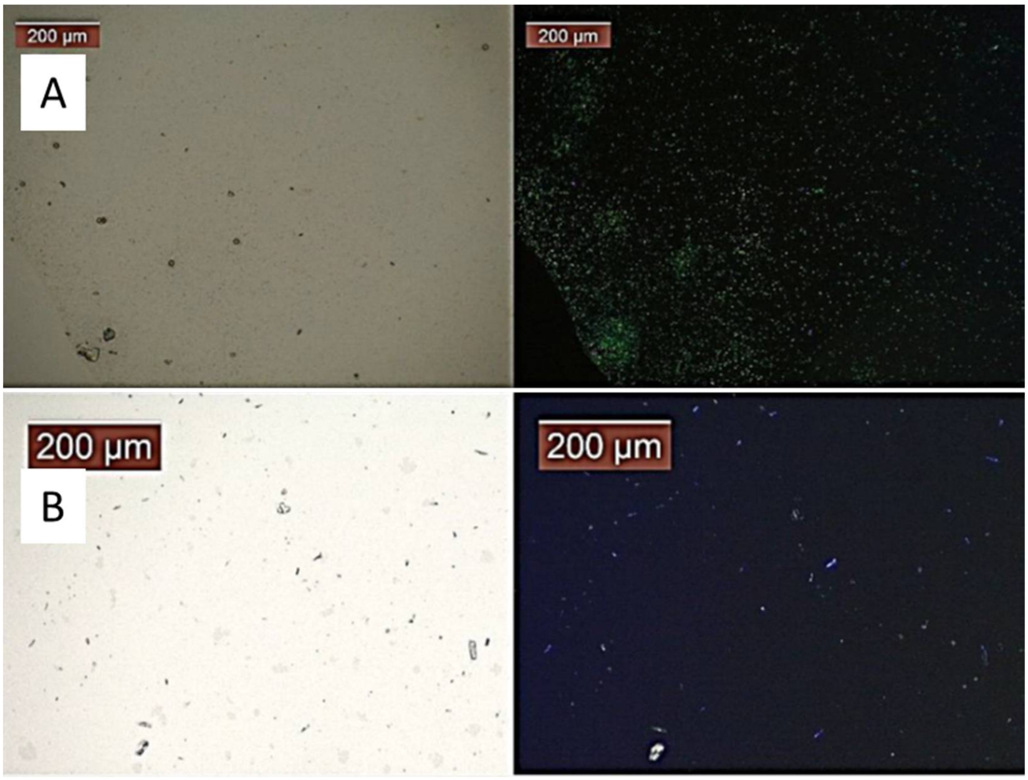

Figure S15. Optical microscopy images of **1** (A) and **2** (B) in DMSO at c.a.c. Right images were recorded with crossed polarizers.

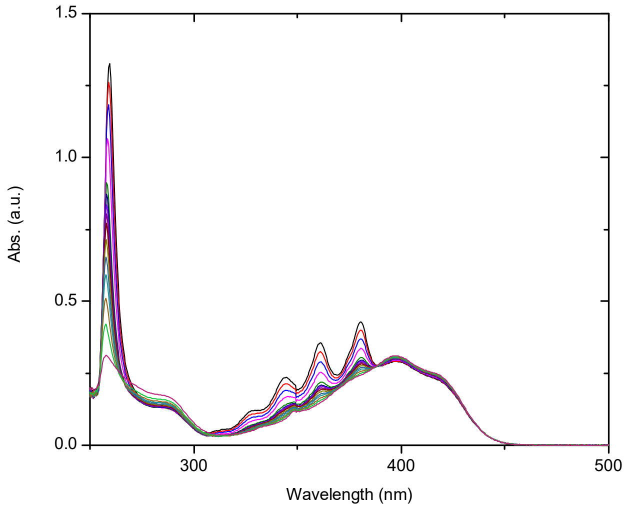

Figure S16. Absorption spectra of **1** in the presence of different amounts of anthracene.

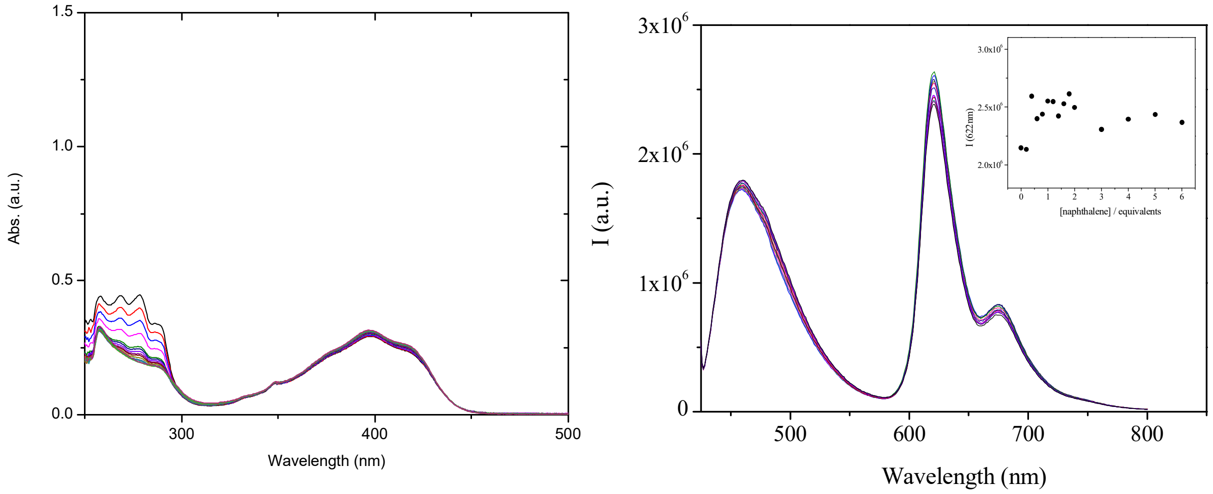

Figure S17. Absorption (left) and Emission (right) spectra of **1** in the presence of different amounts of naphthalene. Inset: variations of the emission maxima at 622 nm against [naphthalene].

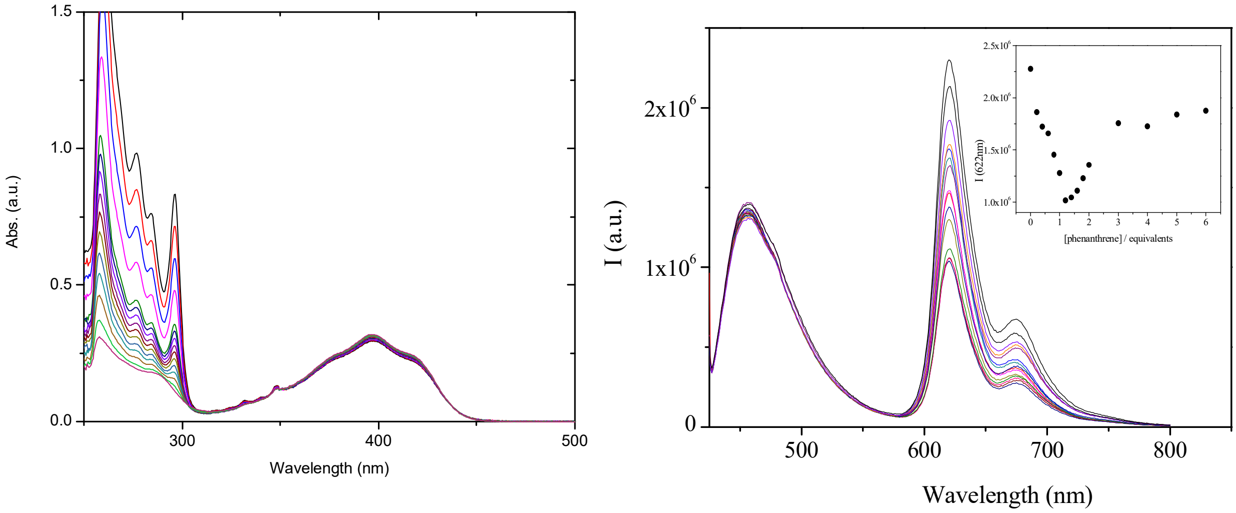

Figure S18. Absorption (left) and Emission (right) spectra of **1** in the presence of different amounts of phenanthrene. Inset: variations of the emission maxima at 622 nm against [phenanthrene].

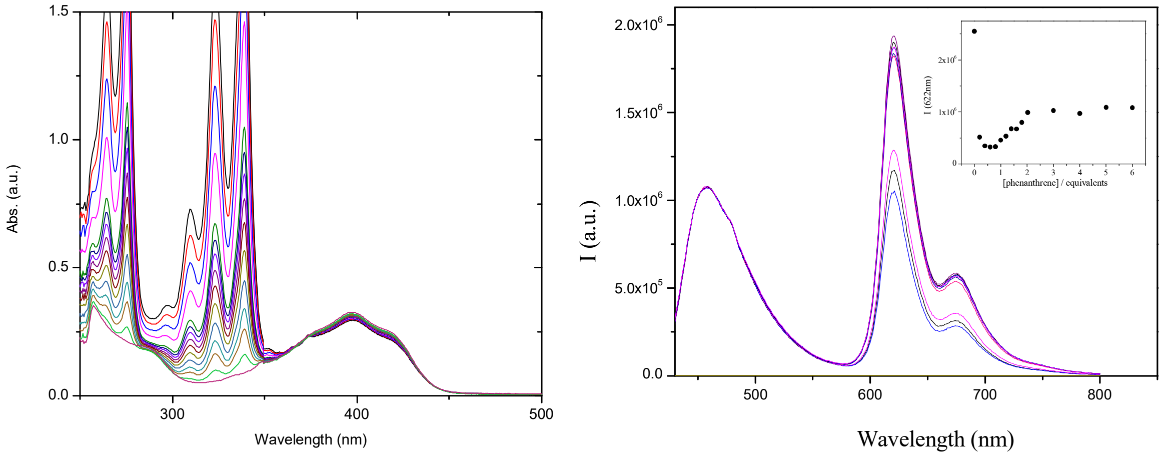

Figure S19. Absorption (left) and Emission (right) spectra of **1** in the presence of different amounts of pyrene. Inset: variations of the emission maxima at 622 nm against [pyrene].

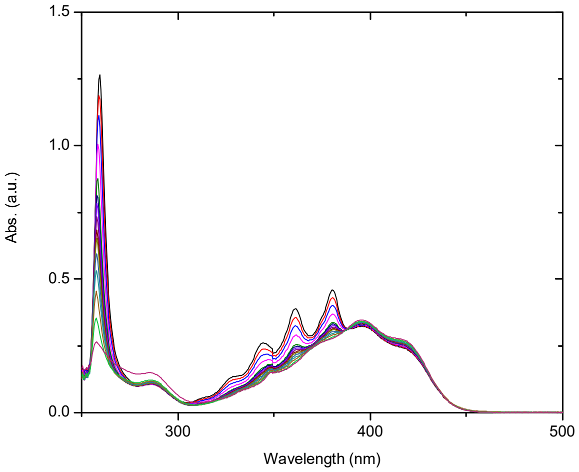

Figure S20. Absorption spectra of **2** in the presence of different amounts of anthracene.

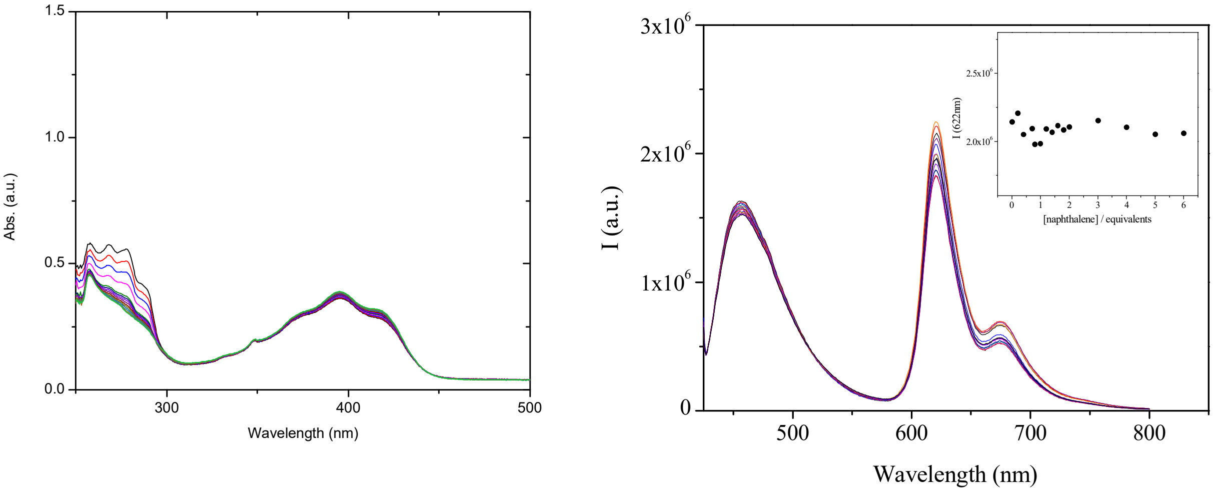

Figure S21. Absorption (left) and Emission (right) spectra of **2** in the presence of different amounts of naphthalene. Inset: variations of the emission maxima at 622 nm against [naphthalene].

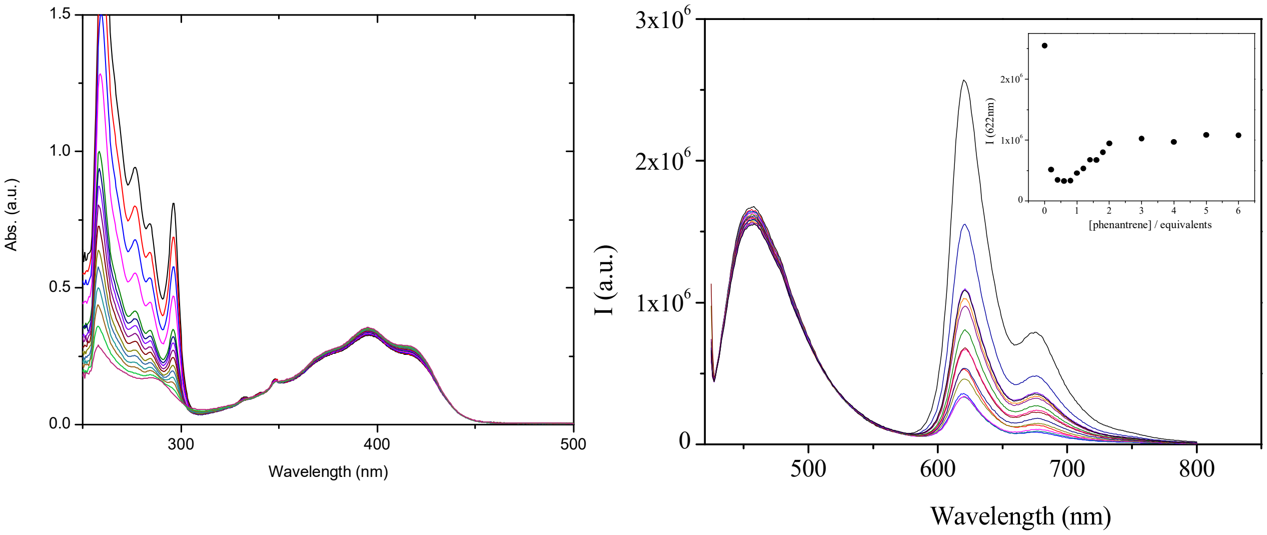

Figure S22. Absorption (left) and Emission (right) spectra of **2** in the presence of different amounts of phenanthrene. Inset: variations of the emission maxima at 622 nm against [phenanthrene].

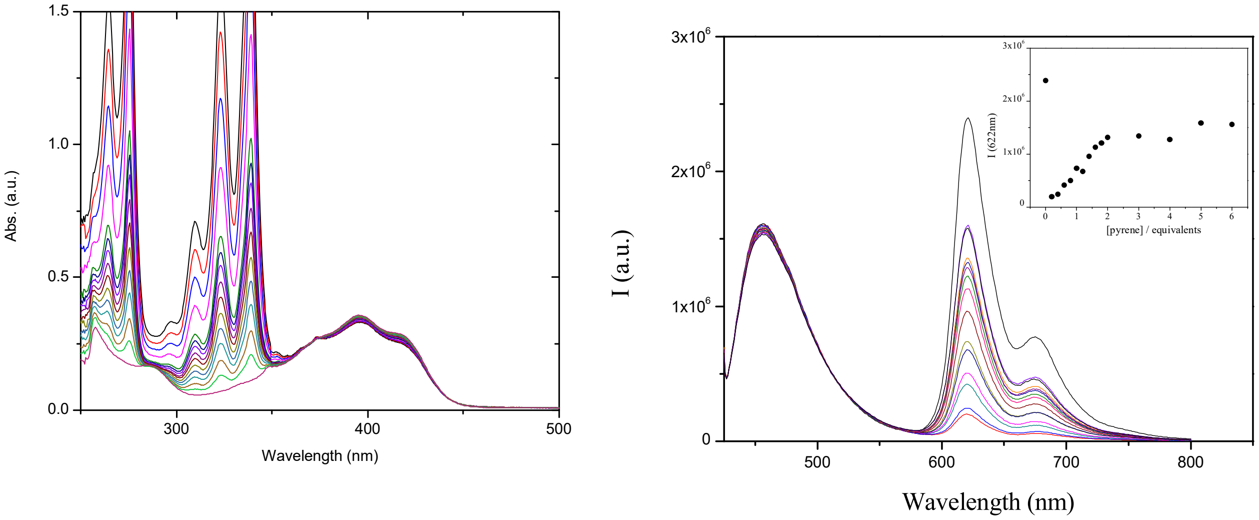

Figure S23. Emission spectra of **2** in the presence of different amounts of pyrene. Inset: variations of the emission maxima at 622 nm against [pyrene].

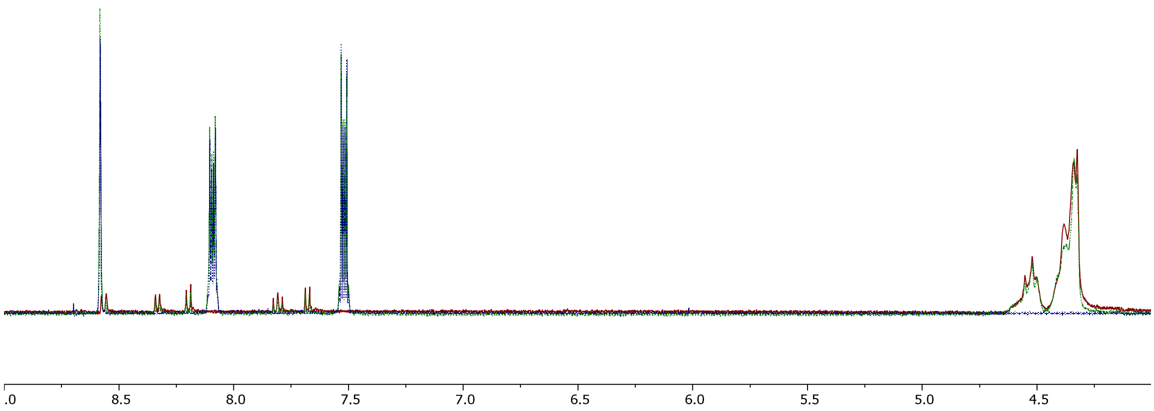

Figure S24. ^{1}H NMR spectra in DMSO-d_6 of **1** (red), anthracene (blue) and 1:1 adduct **1**:anthracene (green)

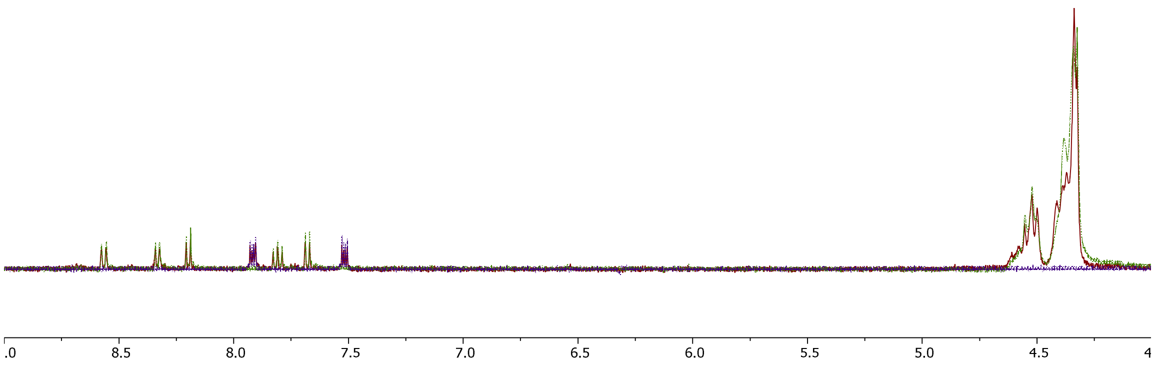

Figure S25. ^{1}H NMR spectra in DMSO-d_6 of **1** (green), naphthalene (purple) and 1:1 adduct **1**: naphthalene (red)

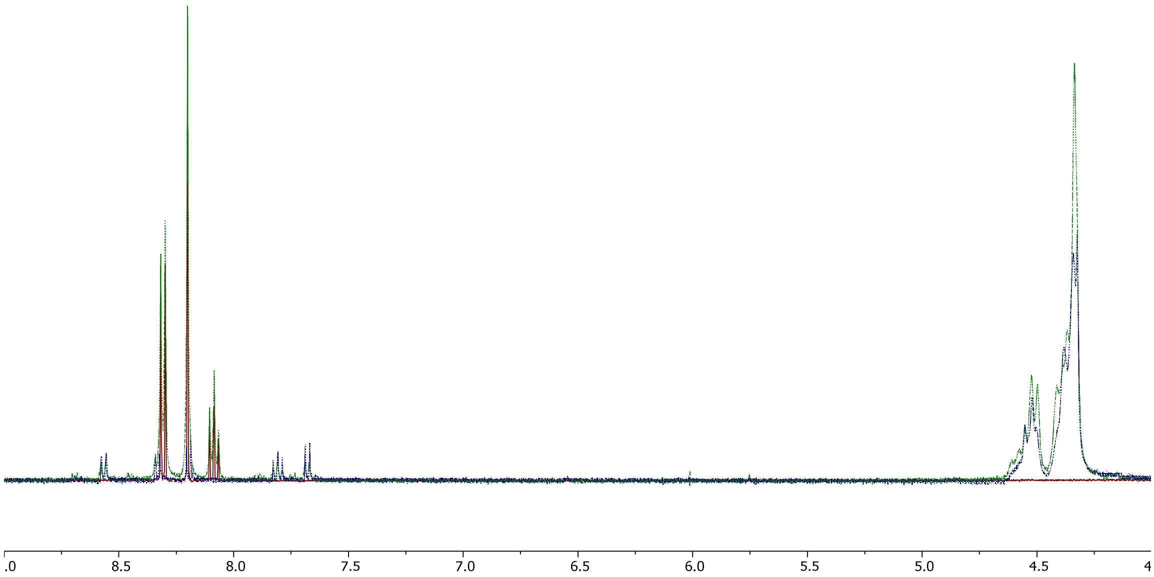

Figure S26. ^{1}H NMR spectra in DMSO-d_6 of **1** (blue), pyrene (red) and 1:1 adduct **1**: pyrene (green)

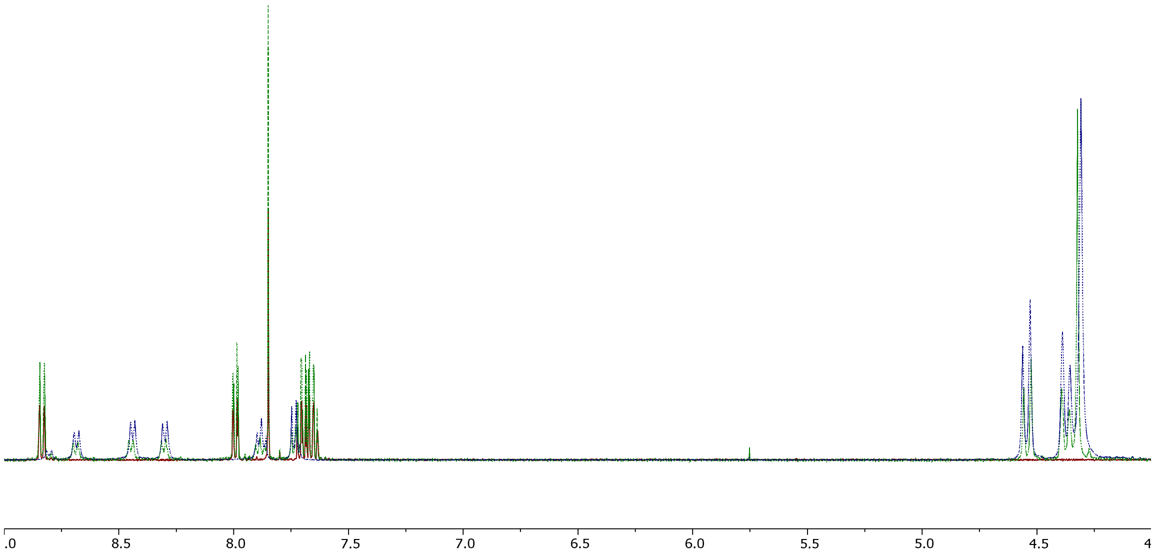

Figure S27. ^{1}H NMR spectra in DMSO-d_6 of **1** (blue), phenanthrene (red) and 1:1 adduct **1**: phenanthrene (green)

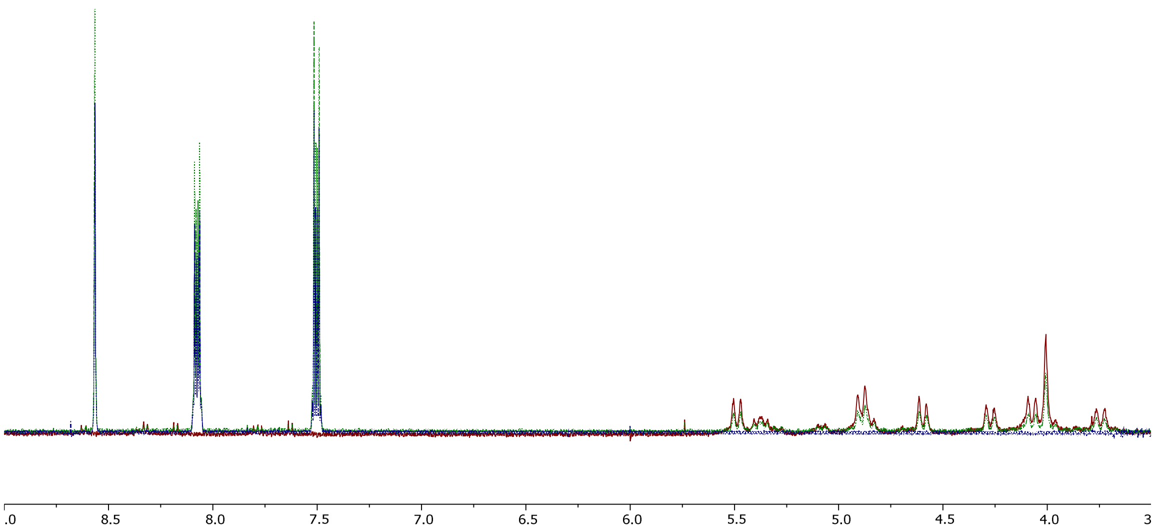

Figure S28. ^{1}H NMR spectra in DMSO-d_6 of **2** (red), anthracene (blue) and 1:1 adduct **2**:anthracene (green)

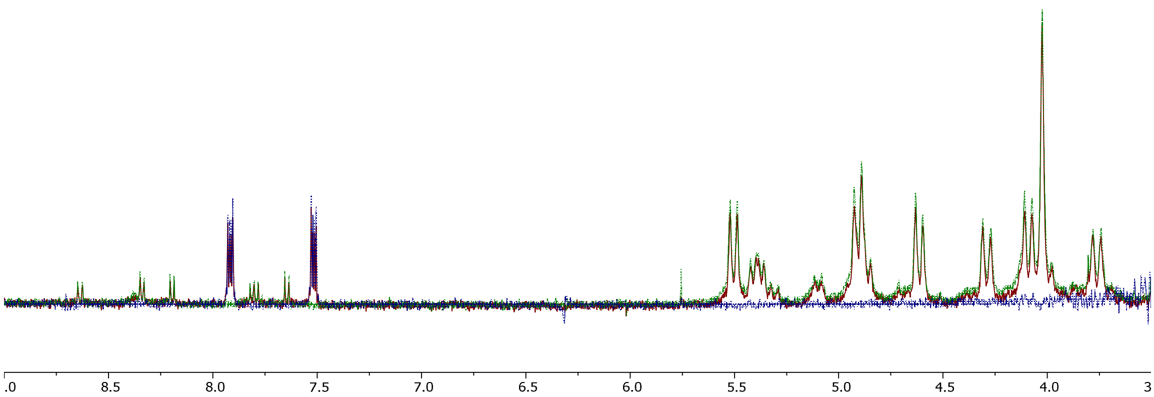

Figure S29. ^{1}H NMR spectra in DMSO-d_6 of **2** (green), naphthalene (blue) and 1:1 adduct **2**: naphthalene (red)

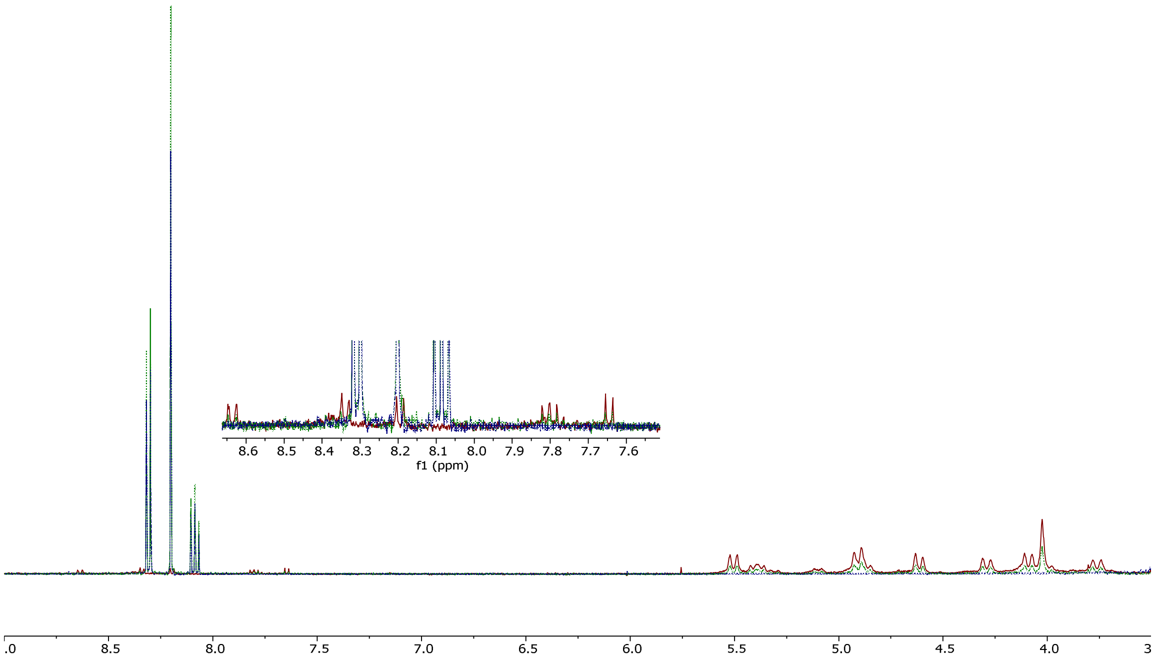

Figure S30. ^{1}H NMR spectra in DMSO-d_6 of **2** (red), pyrene (blue) and 1:1 adduct **2**: pyrene (green)

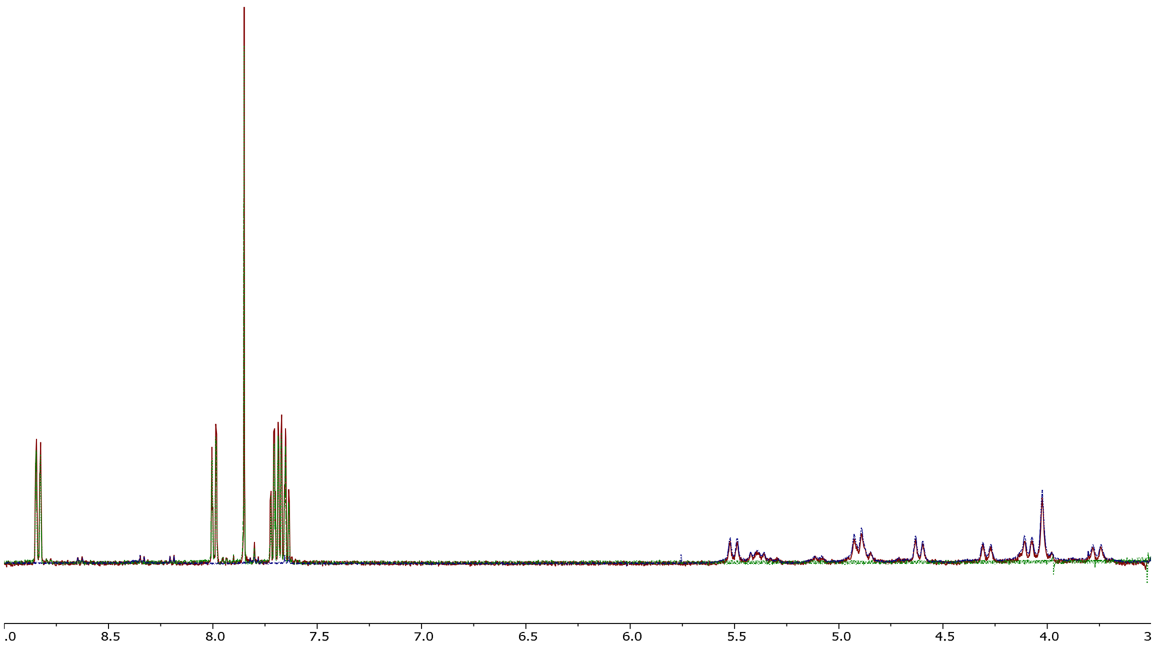

Figure S31. ^{1}H NMR spectra in DMSO-d_6 of **2** (blue), phenantrene (green) and 1:1 adduct **2**: phenantrene (red)

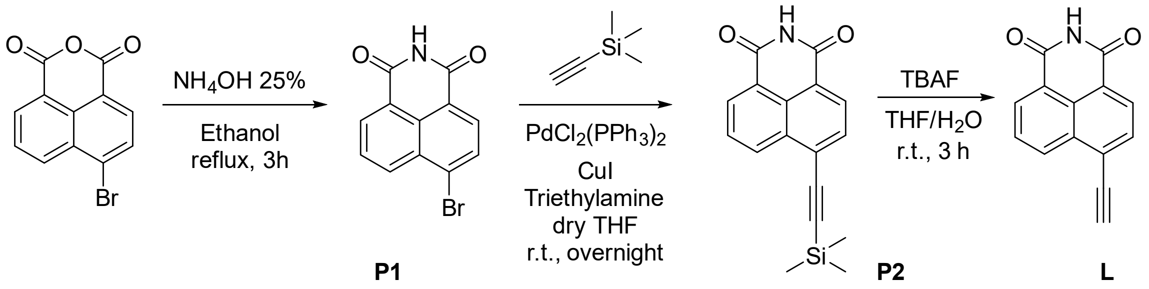

Scheme S1. Experimental procedure for the synthesis of **L**.

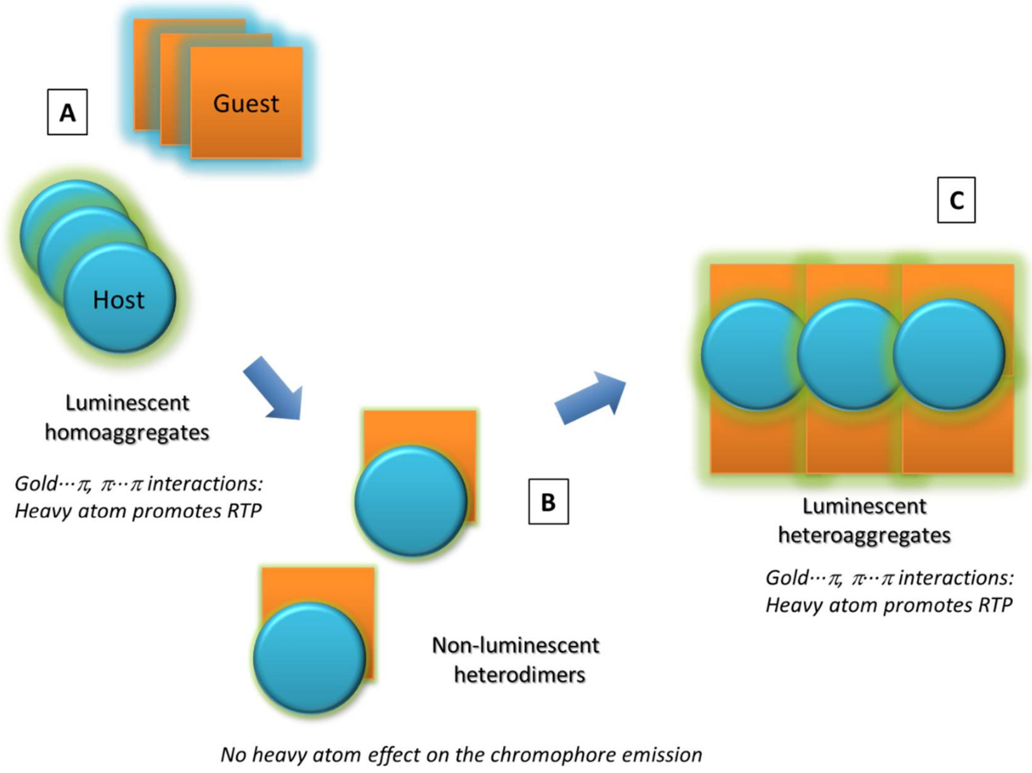

Scheme S2. Schematic representation of the possible rationalization of the different steps of the host:guest interaction and resulting aggregates.
